# Supplementary material for: In silico co-factor balance estimation using constraint-based modelling informs metabolic engineering in Escherichia coli
Source: PLoS Comput Biol. 2020 Aug 10;16(8):e1008125. doi: 10.1371/journal.pcbi.1008125 (PMC7440669; doi:10.1371/journal.pcbi.1008125)
Supplement: S1 Table — Used 100% of optimum and optimized for biomass formation or production of butanol, crotonate, butyric acid or butyraldehyde, accordingly. Minimal and maximal range units are in mmol gDW-1 hr-1. Highlighted in grey–reactions presenting variability ranges, instead of unique fluxes. (DOCX) [file pcbi.1008125.s001.docx]

| **Table S1 \| Flux Variability Analysis of the Wild Type and engineered models using the *Escherichia coli* Core Model.** Optimized for biomass formation (WT) or butanol production (all others), accordingly.. Minimal and maximal range units are in mmol gDW^-1^ hr^-1^. Highlighted in grey – reactions presenting variability ranges, instead of unique fluxes. | | | | | | | | | | | | |
| --- | --- | --- | --- | --- | --- | --- | --- | --- | --- | --- | --- | --- |
|  | **WT** | | **BuOH-0** | | **BuOH-1** | | **tpcBuOH** | | **BuOH-2** | | **fasBuOH** | |
|  | min | max | min | max | min | max | min | max | min | max | min | max |
| NPHT7 |  |  |  |  | 10.0 | 10.0 |  |  | 9.049 | 9.049 |  |  |
| G6PDH2r | 4.72 | 4.72 |  |  |  |  | 2.53 | 2.53 | 4.288 | 4.288 | 6.265 | 6.265 |
| PGM | -14.85 | -14.85 | -20.0 | -20.0 | -20.0 | -20.0 | -19.16 | -19.16 | -18.571 | -18.571 | -17.912 | -17.912 |
| FBP |  |  |  | 12.40 |  | 2.40 |  |  |  |  |  |  |
| FUM | 5.34 | 5.34 |  |  |  |  |  |  | 0.473 | 0.473 |  |  |
| Biomass | 0.86 | 0.86 |  |  |  |  |  |  |  |  |  |  |
| PGI | 5.11 | 5.11 | 10.0 | 10.0 | 10.0 | 10.0 | 7.47 | 7.47 | 5.712 | 5.712 | 3.735 | 3.735 |
| GND | 4.72 | 4.72 | 10.0 | 10.0 |  |  | 2.53 | 2.53 | 4.288 | 4.288 | 6.265 | 6.265 |
| NADH11 | 39.36 | 39.36 |  |  |  |  | 5.07 | 5.07 | 10.941 | 10.941 | 12.530 | 12.530 |
| PPC | 2.47 | 2.47 |  | 12.40 |  | 2.40 |  |  |  |  |  |  |
| MDH | 5.34 | 5.34 | -11.20 |  | -2.40 |  |  |  | 0.473 | 0.473 |  |  |
| PPCK |  |  |  | 12.40 |  | 2.40 |  |  |  |  |  |  |
| PPS |  |  |  | 12.40 |  | 2.40 |  |  |  |  |  |  |
| ME1 |  |  |  | 11.2 |  | 2.40 |  |  |  |  |  |  |
| ME2 |  |  |  | 11.2 |  | 2.40 |  |  |  |  |  |  |
| AKGDH | 5.34 | 5.34 |  |  |  |  |  |  | 0.473 | 0.473 |  |  |
| PGK | -16.14 | -16.14 | -20.0 | -20.0 | -20.0 | -20.0 | -19.16 | -19.16 | -18.571 | -18.571 | -17.912 | -17.912 |
| ADK1 |  |  |  | 12.40 |  | 2.40 | 9.58 | 9.58 | 9.049 | 9.049 | 8.956 | 8.956 |
| EX_glc_e_ | -10.0 | -10.0 | -10.0 | -10.0 | -10.0 | -10.0 | -10.0 | -10.0 | -10.0 | -10.0 | -10.0 | -10.0 |
| ATPM | 7.60 | 7.60 | 7.60 | 20.0 | 7.60 | 10.0 | 7.60 | 7.60 | 7.600 | 7.60 | 7.60 | 7.60 |
| RPI | -2.19 | -2.19 |  |  |  |  | -0.84 | -0.84 | -1.429 | -1.429 | -2.088 | -2.088 |
| RPE | 2.53 | 2.53 |  |  |  |  | 1.69 | 1.69 | 2.859 | 2.859 | 4.177 | 4.177 |
| TALA | 1.42 | 1.42 |  |  |  |  | 0.84 | 0.84 | 1.429 | 1.429 | 2.088 | 2.088 |
| SUCD4 | 5.34 | 5.34 |  |  |  |  |  |  | 0.473 | 0.473 |  |  |
| ICDHyr | 6.26 | 6.26 |  |  |  |  |  |  | 0.473 | 0.473 |  |  |
| GAPD | 16.14 | 16.14 | 20.0 | 20.0 | 20.0 | 20.0 | 19.16 | 19.16 | 18.571 | 18.571 | 17.912 | 17.912 |
| CYTBD | 44.69 | 44.69 |  |  |  |  | 5.07 | 5.07 | 11.415 | 11.415 | 12.530 | 12.530 |
| SUCOAS | -5.34 | -5.34 |  |  |  |  |  |  | -0.473 | -0.473 |  |  |
| ENO | 14.85 | 14.85 | 20.0 | 20.0 | 20.0 | 20.0 | 19.16 | 19.16 | 18.571 | 18.571 | 17.912 | 17.912 |
| PDH | 9.49 | 9.49 | 20.0 | 20.0 | 20.0 | 20.0 | 19.16 | 19.16 | 18.571 | 18.571 | 17.912 | 17.912 |
| CS | 6.26 | 6.26 |  |  |  |  |  |  | 0.473 | 0.473 |  |  |
| ATPS4r | 39.75 | 39.75 | -12.40 |  | -2.40 |  | 7.60 | 7.60 | 15.702 | 15.702 | 16.556 | 16.556 |
| TKT2 | 1.11 | 1.11 |  |  |  |  | 0.84 | 0.84 | 1.429 | 1.429 | 2.088 | 2.088 |
| TKT1 | 1.42 | 1.42 |  |  |  |  | 0.84 | 0.84 | 1.429 | 1.429 | 2.088 | 2.088 |
| PGL | 4.72 | 4.72 |  |  |  |  | 2.53 | 2.53 | 4.288 | 4.288 | 6.265 | 6.265 |
| PFK | 7.57 | 7.57 | 10.00 | 22.40 | 10.0 | 12.40 | 9.16 | 9.16 | 8.571 | 8.571 | 7.912 | 7.912 |
| FBA | 7.57 | 7.57 | 10.0 | 10.0 | 10.0 | 10.0 | 9.16 | 9.16 | 8.571 | 8.571 | 7.912 | 7.912 |
| PYK | 1.93 | 1.93 |  | 22.40 | 7.60 | 12.40 | 9.16 | 9.16 | 8.571 | 8.571 | 7.912 | 7.912 |
| ACONT | 6.26 | 6.26 | 10.0 | 10.0 |  |  |  |  | 0.473 | 0.473 |  |  |
| TPI | 7.57 | 7.57 |  |  | 10.0 | 10.0 | 9.16 | 9.16 | 8.571 | 8.571 | 7.912 | 7.912 |
| NADTRHD |  |  |  | 24.8 |  | 4.8 |  |  |  |  |  |  |
| THD2 |  |  |  | 24.8 |  | 4.8 | 4.51 | 4.51 |  |  | 5.382 | 5.382 |
| HCO3E |  |  |  |  | 10.0 | 10.0 |  |  | 9.049 | 9.049 | 8.956 | 8.956 |
| ACCOAC |  |  |  |  | 10.0 | 10.0 |  |  | 9.049 | 9.049 | 8.956 | 8.956 |
| BUT1 |  |  | 10.0 | 10.0 |  |  | 9.58 | 9.58 |  |  |  |  |
| BUT2 |  |  | 10.0 | 10.0 | 10.0 | 10.0 | 9.58 | 9.58 | 9.049 | 9.049 |  |  |
| BUT3 |  |  | 10.0 | 10.0 | 10.0 | 10.0 | 9.58 | 9.58 | 9.049 | 9.049 |  |  |
| BUT4 |  |  | 10.0 | 10.0 | 10.0 | 10.0 | 9.58 | 9.58 | 9.049 | 9.049 |  |  |
| BUT5 |  |  | 10.0 | 10.0 | 10.0 | 10.0 |  |  |  |  |  |  |
| BUT6 |  |  | 10.0 | 10.0 | 10.0 | 10.0 | 9.58 | 9.58 | 9.049 | 9.049 | 8.956 | 8.956 |
| CAR |  |  |  |  |  |  | 9.58 | 9.58 | 9.049 | 9.049 | 8.956 | 8.956 |
| BTBTAC |  |  |  |  |  |  | 9.58 | 9.58 | 9.049 | 9.049 |  |  |
| BTOH_tr |  |  | 10.0 | 10.0 | 10.0 | 10.0 | 9.58 | 9.58 | 9.049 | 9.049 | 8.956 | 8.956 |
| BTOH_sink |  |  | 10.0 | 10.0 | 10.0 | 10.0 | 9.58 | 9.58 | 9.049 | 9.049 | 8.956 | 8.956 |
| 3HAD40 |  |  |  |  |  |  |  |  |  |  | 8.956 | 8.956 |
| BPNT |  |  |  |  |  |  |  |  |  |  |  |  |
| MCOATA |  |  |  |  |  |  |  |  |  |  | 8.956 | 8.956 |
| 3OAR40 |  |  |  |  |  |  |  |  |  |  | 8.956 | 8.956 |
| EAR40x |  |  |  |  |  |  |  |  |  |  | 8.956 | 8.956 |
| KAS15 |  |  |  |  |  |  |  |  |  |  | 8.956 | 8.956 |
| 5_BUT1 |  |  |  |  |  |  |  |  |  |  | 8.956 | 8.956 |

| **Table S1 (continued) \| Flux Variability Analysis of the butanol precursor models using the *Escherichia coli* Core Model.** Used 100% of optimum and optimized for crotonate, butyric acid or butyraldehyde production, accordingly. Minimal and maximal range units are mmol gDW^-1^ hr^-1^ | | | | | | |
| --- | --- | --- | --- | --- | --- | --- |
|  | **CROT** | | **BUTYR** | | **BUTAL** | |
|  | min | max | min | max | min | max |
| PGM | -20.0 | -20.0 | -20.0 | -20.0 | -20.0 | -20.0 |
| PFL |  | 20.0 |  | 20.0 |  | 10.0 |
| GLCpts | 10.0 | 10.0 | 10.0 | 10.0 | 10.0 | 10.0 |
| PGI | 10.0 | 10.0 | 10.0 | 10.0 | 10.0 | 10.0 |
| FBP |  | 42.40 |  | 32.40 |  | 22.40 |
| NADH11 | 10.0 | 30.0 |  | 20.00 |  | 10.0 |
| MDH | -26.20 |  | -21.20 |  | -16.20 |  |
| ME1 |  | 26.20 |  | 21.20 |  | 16.20 |
| ME2 |  | 26.20 |  | 21.20 |  | 16.20 |
| PGK | -20.0 | -20.0 | -20.0 | -20.0 | -20.00 | -20.0 |
| PPC |  | 42.4 |  | 32.40 |  | 22.40 |
| PPCK |  | 42.4 |  | 32.40 |  | 22.40 |
| ADK1 |  | 42.40 |  | 32.40 |  | 22.40 |
| EX_glc_e_ | -10.0 | -10.0 | -10.0 | -10.0 | -10.0 | -10.0 |
| ATPM | 7.60 | 50.0 | 7.60 | 40.0 | 7.60 | 30.0 |
| GAPD | 20.0 | 20.0 | 20.0 | 20.0 | 20.0 | 20.0 |
| CYTBD | 10.0 | 30.0 |  | 20.0 |  | 10.0 |
| ENO | 20.0 | 20.0 | 20.0 | 20.0 | 20.0 | 20.0 |
| PDH |  | 20.0 |  | 20.0 | 10.0 | 20.0 |
| PPS |  | 42.40 |  | 32.40 |  | 22.40 |
| PYK |  | 52.40 |  | 42.40 |  | 32.40 |
| ATPS4r | -12.40 | 30.0 | -12.40 | 20.0 | -12.40 | 10.0 |
| PFK | 10.0 | 52.40 | 10.0 | 42.40 | 10.0 | 32.40 |
| FBA | 10.0 | 10.0 | 10.0 | 10.0 | 10.0 | 10.0 |
| TPI | 10.0 | 10.0 | 10.0 | 10.0 | 10.0 | 10.0 |
| NADTRHD |  | 84.8 |  | 64.8 |  | 44.8 |
| THD2 |  | 84.80 |  | 64.8 |  | 44.8 |
| BUT1 | 10.0 | 10.0 | 10.0 | 10.0 | 10.0 | 10.0 |
| BUT2 | 10.0 | 10.0 | 10.0 | 10.0 | 10.0 | 10.0 |
| BUT3 | 10.0 | 10.0 | 10.0 | 10.0 | 10.0 | 10.0 |
| BUT4 |  |  | 10.0 | 10.0 | 10.0 | 10.0 |
| BUT5 |  |  |  |  | 10.0 | 10.0 |
| BTBTAC |  |  | 10.0 | 10.0 |  |  |
| B2CTCRO | 10.0 | 10.0 |  |  |  |  |
| CROAC_tr | 10.0 | 10.0 |  |  |  |  |
| CROAC_sink | 10.0 | 10.0 |  |  |  |  |
| BTAC_tr |  |  | 10.0 | 10.0 |  |  |
| BTAC_sink |  |  | 10.0 | 10.0 |  |  |
| BTAL_tr |  |  |  |  | 10.0 | 10.0 |
| BTAL_sink |  |  |  |  | 10.0 | 10.0 |
