# Supplementary material for: In silico co-factor balance estimation using constraint-based modelling informs metabolic engineering in Escherichia coli
Source: PLoS Comput Biol. 2020 Aug 10;16(8):e1008125. doi: 10.1371/journal.pcbi.1008125 (PMC7440669; doi:10.1371/journal.pcbi.1008125)
Supplement: S2 Table — Used the Escherichia coli Core Model and parsimonious FBA for optimization, using either biomass formation or target production as the objective function, accordingly. Solutions were simulated under aerobic conditions (EX_o2_e_ = -10 mmol gDW-1 hr-1) and were otherwise unconstrained. (DOCX) [file pcbi.1008125.s002.docx]

| **Table S2 \| pFBA flux distributions of wild type and engineered models under aerobic conditions.** Used the *Escherichia coli* Core Model and parsimonious FBA for optimization, using either biomass formation or target production as the objective function, accordingly. Solutions were simulated under aerobic conditions (EX_o2_e_ = -10 mmol gDW^-1^ hr^-1^ ) and were otherwise unconstrained. | | | | | | | | | |
| --- | --- | --- | --- | --- | --- | --- | --- | --- | --- |
|  | **WT** | **BuOH-0** | **BuOH-1** | **tpcBuOH** | **BuOH-2** | **fasBuOH** | **CROT** | **BUTYR** | **BUTAL** |
| ADK1 |  |  |  | 9.58 | 9.05 | 8.96 |  |  |  |
| ACONT | 6.27 |  |  |  | 0.47 |  |  |  |  |
| AKGDH | 5.34 |  |  |  | 0.47 |  |  |  |  |
| ATPM | 7.60 | 20 | 10 | 7.60 | 7.60 | 7.60 | 25 | 15 | 17.5 |
| ATPS4r | 39.75 |  |  | 7.60 | 15.70 | 16.56 | 5 | -5 | -2.5 |
| Biomass | 0.86 |  |  |  |  |  |  |  |  |
| CS | 6.27 |  |  |  | 0.47 |  |  |  |  |
| CYTBD | 44.69 |  |  | 5.07 | 11.42 | 12.53 | 10 |  |  |
| ENO | 14.85 | 20 | 20 | 19.16 | 18.57 | 17.91 | 20 | 20 | 20 |
| FBA | 7.57 | 10 | 10 | 9.16 | 8.57 | 7.91 | 10 | 10 | 10 |
| FUM | 5.34 |  |  |  | 0.47 |  |  |  |  |
| G6PDH2r | 4.72 |  |  | 2.53 | 4.29 | 6.27 |  |  |  |
| GAPD | 16.14 | 20 | 20 | 19.16 | 18.57 | 17.91 | 20 | 20 | 20 |
| GLCpts | 10.00 | 10 | 10 | 10.00 | 10.00 | 10.00 | 10 | 10 | 10 |
| GND | 4.72 |  |  | 2.53 | 4.29 | 6.27 |  |  |  |
| ICDHyr | 6.27 |  |  |  | 0.47 |  |  |  |  |
| MDH | 5.34 |  |  |  | 0.47 |  |  |  |  |
| NADH11 | 39.36 |  |  | 5.07 | 10.94 | 12.53 | 10 |  |  |
| PDH | 9.49 | 20 | 20 | 19.16 | 18.57 | 17.91 |  |  | 10 |
| PFK | 7.57 | 10 | 10 | 9.16 | 8.57 | 7.91 | 10 | 10 | 10 |
| PFL |  |  |  |  |  |  | 20 | 20 | 10 |
| PGI | 5.11 | 10 | 10 | 7.47 | 5.71 | 3.74 | 10 | 10 | 10 |
| PGK | -16.14 | -20 | -20 | -19.16 | -18.57 | -17.91 | -20 | -20 | -20 |
| PGL | 4.72 |  |  | 2.53 | 4.29 | 6.27 |  |  |  |
| PGM | -14.85 | -20 | -20 | -19.16 | -18.57 | -17.91 | -20 | -20 | -20 |
| PPC | 2.47 |  |  |  |  |  |  |  |  |
| PYK | 1.93 | 10 | 10 | 9.16 | 8.57 | 7.91 | 10 | 10 | 10 |
| RPE | 2.53 |  |  | 1.69 | 2.86 | 4.18 |  |  |  |
| RPI | -2.19 |  |  | -0.84 | -1.43 | -2.09 |  |  |  |
| SUCD1i | 5.34 |  |  |  | 0.47 |  |  |  |  |
| SUCD4 | 5.34 |  |  |  | 0.47 |  |  |  |  |
| SUCOAS | -5.34 |  |  |  | -0.47 |  |  |  |  |
| TALA | 1.42 |  |  | 0.84 | 1.43 | 2.09 |  |  |  |
| THD2 |  |  |  | 4.51 |  | 5.38 |  |  |  |
| TKT1 | 1.42 |  |  | 0.84 | 1.43 | 2.09 |  |  |  |
| TKT2 | 1.11 |  |  | 0.84 | 1.43 | 2.09 |  |  |  |
| TPI | 7.57 | 10 | 10 | 9.16 | 8.57 | 7.91 | 10 | 10 | 10 |
| BUT1 |  | 10 |  | 9.58 |  |  | 10 | 10 | 10 |
| BUT2 |  | 10 | 10 | 9.58 | 9.05 |  | 10 | 10 | 10 |
| BUT3 |  | 10 | 10 | 9.58 | 9.05 |  | 10 | 10 | 10 |
| BUT4 |  | 10 | 10 | 9.58 | 9.05 |  |  | 10 | 10 |
| BUT5 |  | 10 | 10 |  |  |  |  |  | 10 |
| BUT6 |  | 10 | 10 | 9.58 | 9.05 | 8.96 |  |  |  |
| BTOH_tr |  | 10 | 10 | 9.58 | 9.05 | 8.96 |  |  |  |
| BTOH_sink |  | 10 | 10 | 9.58 | 9.05 | 8.96 |  |  |  |
| HCO3E |  |  | 10 |  | 9.05 | 8.96 |  |  |  |
| ACCOAC |  |  | 10 |  | 9.05 | 8.96 |  |  |  |
| NPHT7 |  |  | 10 |  | 9.05 |  |  |  |  |
| BTBTAC |  |  |  | 9.58 | 9.05 |  |  | 10 |  |
| CAR |  |  |  | 9.58 | 9.05 | 8.96 |  |  |  |
| MCOATA |  |  |  |  |  | 8.96 |  |  |  |
| KAS15 |  |  |  |  |  | 8.96 |  |  |  |
| 3OAR40 |  |  |  |  |  | 8.96 |  |  |  |
| 3HAD40 |  |  |  |  |  | 8.96 |  |  |  |
| EAR40x |  |  |  |  |  | 8.96 |  |  |  |
| 5_BUT1 |  |  |  |  |  | 8.96 |  |  |  |
| B2CTCRO |  |  |  |  |  |  | 10 |  |  |
| CROAC_tr |  |  |  |  |  |  | 10 |  |  |
| CROT_sink |  |  |  |  |  |  | 10 |  |  |
| BTAC_tr |  |  |  |  |  |  |  | 10 |  |
| BTAC_sink |  |  |  |  |  |  |  | 10 |  |
| BTAL_tr |  |  |  |  |  |  |  |  | 10 |
| BTAL_sink |  |  |  |  |  |  |  |  | 10 |
