# Supplementary material for: In silico co-factor balance estimation using constraint-based modelling informs metabolic engineering in Escherichia coli
Source: PLoS Comput Biol. 2020 Aug 10;16(8):e1008125. doi: 10.1371/journal.pcbi.1008125 (PMC7440669; doi:10.1371/journal.pcbi.1008125)
Supplement: S3 Table — Used the Escherichia coli Core Model and parsimonious FBA for optimization, using either biomass formation or target production as the objective function, accordingly. Solutions were simulated under anaerobic conditions (EX_o2_e_ = 0 mmol gDW-1 hr-1) and were otherwise unconstrained. (DOCX) [file pcbi.1008125.s003.docx]

| **Table S3 \| pFBA flux distributions of wild type and engineered models under anaerobic conditions.** Used the Escherichia coli Core Model and parsimonious FBA for optimization, using either biomass formation or target production as the objective function, accordingly. Solutions were simulated under anaerobic conditions (EX_o2_e_ = 0 mmol gDW^-1^ hr^-1^ ) and were otherwise unconstrained. | | | | | | | | | |  |
| --- | --- | --- | --- | --- | --- | --- | --- | --- | --- | --- |
|  | **WT** | **BuOH-0** | **BuOH-1** | **tpcBuOH** | **BuOH-2** | **fasBuOH** | **CROT** | **BUTYR** | **BUTAL** | |
| Biomass | 0.241 |  |  |  |  |  |  |  |  | |
| G6PDH2r |  |  |  | 3.733 | 1.440 | 0.649 |  |  |  | |
| ADK1 |  |  |  | 7.467 | 5.600 | 5.271 |  |  |  | |
| NPHT7 |  |  | 10.0 |  | 5.600 |  |  |  |  | |
| ATPM | 7.600 | 20.0 | 10.0 | 7.60 | 7.60 | 7.600 | 16.667 | 15.0 | 17.5 | |
| RPE | -0.173 |  |  | 2.489 | 0.960 | 0.433 |  |  |  | |
| GAPD | 19.360 | 20.0 | 20.0 | 18.756 | 19.520 | 19.784 | 20.0 | 20.0 | 20.0 | |
| PPC | 0.690 |  |  |  |  |  |  |  |  | |
| TPI | 9.760 | 10.0 | 10.0 | 8.756 | 9.520 | 9.784 | 10.0 | 10.0 | 10.0 | |
| TKT2 | -0.130 |  |  | 1.244 | 0.480 | 0.216 |  |  |  | |
| TKT1 | -0.043 |  |  | 1.244 | 0.480 | 0.216 |  |  |  | |
| PYK | 8.184 | 10.0 | 10.0 | 8.756 | 9.520 | 9.784 | 10.0 | 10.0 | 10.0 | |
| BTBTAC |  |  |  | 7.467 | 5.600 |  |  | 10.0 |  | |
| ENO | 19.0 | 20.0 | 20.0 | 18.756 | 19.520 | 19.784 | 20.0 | 20.0 | 20.0 | |
| PTAr | 8.297 |  |  | 3.822 | 8.320 | 9.242 |  |  |  | |
| FBA | 9.760 | 10.0 | 10.0 | 8.756 | 9.520 | 9.784 | 10.0 | 10.0 | 10.0 | |
| H2Ot | 8.032 | -10.0 | -10.0 | -14.844 | -7.040 | -5.920 |  |  | -10.0 | |
| ACONT | 0.260 |  |  |  |  |  |  |  |  | |
| HCO3E |  |  | 10.0 |  | 5.60 | 5.271 |  |  |  | |
| TALA | -0.043 |  |  | 1.244 | 0.480 | 0.216 |  |  |  | |
| THD2 | 4.129 |  |  |  | 2.720 | 9.242 |  |  |  | |
| ACKr | -8.297 |  |  | -3.822 | -8.320 | -9.242 |  |  |  | |
| BTOH_sink |  | 10.0 | 10.0 | 7.467 | 5.60 | 5.271 |  |  |  | |
| ICDHyr | 0.260 |  |  |  |  |  |  |  |  | |
| PDH |  | 20.0 | 20.0 | 3.644 |  |  |  |  | 10.0 | |
| CS | 0.260 |  |  |  |  |  |  |  |  | |
| ATPS4r | -5.066 |  |  | -0.044 | -3.440 | -5.614 | -3.333 | -5.0 | -2.5.0 | |
| BUT3 |  | 10.0 | 10.0 | 7.467 | 5.60 |  | 6.667 | 10.0 | 10.0 | |
| ADHEr | 8.042 |  |  |  |  |  | 6.667 |  |  | |
| BUT2 |  | 10.0 | 10.0 | 7.467 | 5.60 |  | 6.667 | 10.0 | 10.0 | |
| BUT1 |  | 10.0 |  | 7.467 |  |  | 6.667 | 10.0 |  | |
| BTOH_tr |  | 10.0 | 10.0 | 7.467 | 5.60 | 5.271 |  |  |  | |
| ACt2r | -8.297 |  |  | -3.822 | -8.320 | -9.242 |  |  |  | |
| BUT6 |  | 10.0 | 10.0 | 7.467 | 5.600 | 5.271 |  |  |  | |
| BUT5 |  | 10.0 | 10.0 |  |  |  |  |  | 10.0 | |
| BUT4 |  | 10.0 | 10.0 | 7.467 | 5.60 |  |  | 10. | 10.0 | |
| CAR |  |  |  | 7.467 | 5.60 | 5.271 |  |  |  | |
| GLCpts | 10.0 | 10.0 | 10.0 | 10.0 | 10.0 | 10.000 | 10.0 | 10.0 | 10.0 | |
| GND |  |  |  | 3.733 | 1.440 | 0.649 |  |  |  | |
| PGL |  |  |  | 3.733 | 1.440 | 0.649 |  |  |  | |
| PGM | -19.0 | -20.0 | -20.0 | -18.756 | -19.520 | -19.784 | -20.0 | -20.0 | -20.0 | |
| PGK | -19.360 | -20.0 | -20.0 | -18.756 | -19.520 | -19.784 | -20.0 | -20.0 | -20.0 | |
| PGI | 9.951 | 10.0 | 10.0 | 6.267 | 8.560 | 9.351 | 10.0 | 10.0 | 10.0 | |
| RPI | -0.173 |  |  | -1.244 | -0.480 | -0.216 |  |  |  | |
| PFK | 9.760 | 10.0 | 10.0 | 8.756 | 9.520 | 9.784 | 10.0 | 10.0 | 10.0 | |
| PFL | 17.502 |  |  | 15.111 | 19.520 | 19.784 | 20.0 | 20.0 | 10.0 | |
| ACCOAC |  |  | 10.0 |  | 5.60 |  |  |  |  | |
| MCOATA |  |  |  |  |  | 5.271 |  |  |  | |
| 3HAD40 |  |  |  |  |  | 5.271 |  |  |  | |
| ACCOAC |  |  |  |  |  | 5.271 |  |  |  | |
| KAS15 |  |  |  |  |  | 5.271 |  |  |  | |
| EAR40x |  |  |  |  |  | 5.271 |  |  |  | |
| 5_BUT1 |  |  |  |  |  | 5.271 |  |  |  | |
| 3OAR40 |  |  |  |  |  | 5.271 |  |  |  | |
| FORt | -17.502 |  |  | -15.111 | -19.520 | -19.784 | -20.0 | -20.0 | -10.0 | |
| CROAC_tr |  |  |  |  |  |  | 6.667 |  |  | |
| CROAC_sink |  |  |  |  |  |  | 6.667 |  |  | |
| EX_for_e_ | 17.502 |  |  | 15.111 | 19.520 | 19.784 | 20.0 | 20.0 | 10.0 | |
| B2CTCRO |  |  |  |  |  |  | 6.667 |  |  | |
| BTAC_sink |  |  |  |  |  |  |  | 10.0 |  | |
| BTAC_tr |  |  |  |  |  |  |  | 10.0 |  | |
| BTAL_sink |  |  |  |  |  |  |  |  | 10.0 | |
| BTAL_tr |  |  |  |  |  |  |  |  | 10.0 | |
