# Supplementary material for: In silico co-factor balance estimation using constraint-based modelling informs metabolic engineering in Escherichia coli
Source: PLoS Comput Biol. 2020 Aug 10;16(8):e1008125. doi: 10.1371/journal.pcbi.1008125 (PMC7440669; doi:10.1371/journal.pcbi.1008125)
Supplement: S4 Table — Reaction IDs, relevant co-factor and their stoichiometric coefficient, flux value, balance value and assigned balance category are included. (DOCX) [file pcbi.1008125.s004.docx]

| **Table S4 \| CBA parameters and outputs of unconstrained models under aerobic conditions.** Reaction IDs, relevant co-factor and their stoichiometric coefficient, flux value, balance value and assigned balance category are included. | | | | | | |
| --- | --- | --- | --- | --- | --- | --- |
| **Reaction ID** | **Co-factor** | **Stoich. coefficient** | **Flux Distribution** | **Balance Value** | **Balance Category** |  |
| **Wild Type (WT)** | | | | | | |
| ATPM | ATP | ­-1 | 7.6 | -7.6 | *Waste* |  |
| Biomass | ATP | -55.703 | 0.87 | -47.983 | *Biomass* |  |
| SUCOAS | ATP | -1 | -5.336 | 5.336 | *Production* |  |
| PYK | ATP | 1 | 1.935 | 1.935 | *Production* |  |
| ATPS4r | ATP | 1 | 39.747 | 39.747 | *Production* |  |
| PGK | ATP | -1 | -16.138 | 16.138 | *Production* |  |
| PFK | ATP | -1 | 7.571 | -7.571 | *Maintenance* |  |
| G6PDH2r | NADPH | 1 | 4.717 | 4.717 | *Waste* |  |
| ICDHyr | NADPH | 1 | 6.265 | 6.265 | *Waste* |  |
| AKGDH | NADH | 1 | 5.336 | 5.336 | *Waste* |  |
| GND | NADPH | 1 | 4.717 | 4.717 | *Waste* |  |
| PDH | NADH | 1 | 9.493 | 9.493 | *Waste* |  |
| GAPD | NADH | 1 | 16.138 | 16.138 | *Production* |  |
| Biomass | NADH/NADPH | 3.547/-18.225 | 0.87 | -15.699 | *Biomass* |  |
|  | | | | | | |
| **BuOH-0** | | | | | | |
| PFK | ATP | -1 | 10 | -10 | *Maintenance* |  |
| PGK | ATP | -1 | -20 | 20 | *Production* |  |
| PYK | ATP | 1 | 10 | 10 | *Production* |  |
| ATPM | ATP | -1 | 20 | -20 | *Waste* |  |
| GAPD | NADH | 1 | 20 | 20 | *Production* |  |
| BUT2 | NADH | -1 | 10 | -10 | *Target* |  |
| BUT4 | NADH | -1 | 10 | -10 | *Target* |  |
| BUT5 | NADH | -1 | 10 | -10 | *Target* |  |
| BUT6 | NADH | -1 | 10 | -10 | *Target* |  |
| PDH | NADH | 1 | 20 | 20 | *Waste* |  |
|  | | | | | | |
| **BuOH-1** | | | | | | |
| PFK | ATP | -1 | 10 | -10 | *Maintenance* |  |
| PGK | ATP | -1 | -20 | 20 | *Production* |  |
| PYK | ATP | 1 | 10 | 10 | *Production* |  |
| ATPM | ATP | -1 | 10 | -10 | *Waste* |  |
| ACCOAC | ATP | -1 | 10 | -10 | *Target* |  |
| GAPD | NADH | 1 | 20 | 20 | *Production* |  |
| BUT2 | NADH | -1 | 10 | -10 | *Target* |  |
| BUT4 | NADH | -1 | 10 | -10 | *Target* |  |
| BUT5 | NADH | -1 | 10 | -10 | *Target* |  |
| BUT6 | NADH | -1 | 10 | -10 | *Target* |  |
| PDH | NADH | 1 | 20 | 20 | *Waste* |  |
|  |  |  |  |  |  |  |
| **tpcBuOH** |  |  |  |  |  |  |
| PYK | ATP | 1 | 9.156 | 9.156 | *Production* | |
| ATPS4r | ATP | 1 | 7.6 | 7.6 | *Production* | |
| PGK | ATP | -1 | -19.156 | 19.156 | *Production* | |
| CAR | ATP | -1 | 9.578 | -9.578 | *Target* | |
| PFK | ATP | -1 | 9.156 | -9.156 | *Maintenance* | |
| ATPM | ATP | -1 | 9.578 | -9.578 | *Waste* | |
| ADK1 | ATP | -1 | 9.578 | -9.578 | *Waste* | |
| GAPD | NADH | 1 | 19.156 | 19.156 | *Production* | |
| THD2 | NADPH | 1 | 4.511 | 4.511 | *Production* | |
| G6PDH2r | NADPH | 1 | 2.533 | 2.533 | *Production* | |
| CAR | NADPH | -1 | 9.578 | -9.578 | *Target* | |
| BUT2 | NADH | -1 | 9.578 | -9.578 | *Target* | |
| BUT4 | NADH | -1 | 9.578 | -9.578 | *Target* | |
| BUT6 | NADH | -1 | 9.578 | -9.578 | *Target* | |
| GND | NADPH | 1 | 2.533 | 2.533 | *Waste* | |
| PDH | NADH | 1 | 19.156 | 19.156 | *Waste* | |
| NADH11 | NADH | -1 | 5.067 | -5.067 | *Maintenance* | |
| THD2 | NADH | -1 | 4.511 | -4.511 | *Maintenance* | |
|  |  |  |  |  |  | |
| **BuOH-2** |  |  |  |  |  | |
| SUCOAS | ATP | 1 | 0.473 | 0.473 | *Production* | |
| PYK | ATP | 1 | 8.571 | 8.571 | *Production* | |
| ATPS4r | ATP | 1 | 15.702 | 15.702 | *Production* | |
| PGK | ATP | -1 | -18.571 | 18.571 | *Production* | |
| CAR | ATP | -1 | 9.049 | -9.049 | *Target* | |
| ACCOAC | ATP | -1 | 9.049 | -9.049 | *Target* | |
| PFK | ATP | -1 | 8.571 | -8.571 | *Maintenance* | |
| ATPM | ATP | -1 | 7.6 | -7.6 | *Waste* | |
| ADK1 | ATP | -1 | 9.049 | -9.049 | *Waste* | |
| MDH | NADH | 1 | 0.473 | 0.473 | *Production* | |
| GAPD | NADH | 1 | 18.570 | 18.570 | *Production* | |
| G6PDH2r | NADPH | 1 | 4.288 | 4.288 | *Production* | |
| CAR | NADPH | -1 | 9.049 | -9.049 | *Target* | |
| BUT2 | NADH | -1 | 9.049 | -9.049 | *Target* | |
| BUT4 | NADH | -1 | 9.049 | -9.049 | *Target* | |
| BUT6 | NADH | -1 | 9.049 | -9.049 | *Target* | |
| NADH11 | NADH | -1 | 10.941 | -10.941 | *Maintenance* | |
| ICDHyr | NADH | 1 | 0.473 | 0.473 | *Waste* | |
| AKGDH | NADH | 1 | 0.473 | 0.473 | *Waste* | |
| GND | NADPH | 1 | 4.288 | 4.288 | *Waste* | |
| PDH | NADH | 1 | 18.571 | 18.571 | *Waste* | |
|  |  |  |  |  |  | |
| **fasBuOH** |  |  |  |  |  | |
| PYK | ATP | 1 | 7.912 | 7.912 | *Production* | |
| ATPS4r | ATP | 1 | 16.556 | 16.556 | *Production* | |
| PGK | ATP | -1 | -17.912 | 17.912 | *Production* | |
| CAR | ATP | -1 | 8.956 | -8.956 | *Target* | |
| ACCOAC | ATP | -1 | 8.956 | -8.956 | *Target* | |
| PFK | ATP | -1 | 7.912 | -7.912 | *Maintenance* | |
| ADK1 | ATP | -1 | 8.956 | -8.956 | *Waste* | |
| ATPM | ATP | -1 | 7.6 | -7.6 | *Waste* | |
| G6PDH2r | NADPH | 1 | 6.265 | 6.265 | *Production* | |
| GAPD | NADH | 1 | 17.912 | 17.912 | *Production* | |
| THD2 | NADPH | 1 | 5.381 | 5.381 | *Production* | |
| CAR | NADPH | -1 | 8.956 | -8.956 | *Target* | |
| BUT6 | NADH | -1 | 8.956 | -8.956 | *Target* | |
| 30AR40 | NADPH | -1 | 8.956 | -8.956 | *Maintenance* | |
| EAR40x | NADH | -1 | 8.956 | -8.956 | *Maintenance* | |
| NADH11 | NADH | -1 | 12.530 | -12.530 | *Maintenance* | |
| THD2 | NADH | -1 | 5.381 | -5.381 | *Maintenance* | |
| GND | NADPH | 1 | 6.265 | 6.265 | *Waste* | |
| PDH | NADH | 1 | 17.912 | 17.912 | *Waste* | |
|  |  |  |  |  |  | |
| **CROT** |  |  |  |  |  | |
| PYK | ATP | 1 | 10 | 10 | *Production* | |
| ATPS4r | ATP | 1 | 5 | 5 | *Production* | |
| PGK | ATP | -1 | -20 | 20 | *Production* | |
| PFK | ATP | -1 | 10 | -10 | *Maintenance* | |
| ATPM | ATP | -1 | 25 | -25 | *Waste* | |
| GAPD | NADH | 1 | 20 | 20 | *Production* | |
| BUT2 | NADH | -1 | 10 | -10 | *Target* | |
| NADH11 | NADH | -1 | 10 | -10 | *Maintenance* | |
|  |  |  |  |  |  | |
| **BUTYR** |  |  |  |  |  | |
| PYK | ATP | 1 | 10 | 10 | *Production* | |
| PGK | ATP | -1 | -20 | 20 | *Production* | |
| ATPS4r | ATP | 1 | -5 | -5 | *Maintenance* | |
| PFK | ATP | -1 | 10 | -10 | *Maintenance* | |
| ATPM | ATP | -1 | 15 | -15 | *Waste* | |
| GAPD | NADH | 1 | 20 | 20 | *Production* | |
| BUT2 | NADH | -1 | 10 | -10 | *Target* | |
| BUT4 | NADH | -1 | 10 | -10 | *Target* | |
|  |  |  |  |  |  | |
| **BUTAL** |  |  |  |  |  | |
| PYK | ATP | 1 | 10 | 10 | *Production* | |
| PGK | ATP | -1 | -20 | 20 | *Production* | |
| ATPS4r | ATP | 1 | -2.5 | -2.5 | *Maintenance* | |
| PFK | ATP | -1 | 10 | -10 | *Maintenance* | |
| ATPM | ATP | -1 | 17.5 | -17.5 | *Waste* | |
| GAPD | NADH | 1 | 20 | 20 | *Production* | |
| PDH | NADH | 1 | 20 | 20 | *Waste* | |
| BUT2 | NADH | -1 | 10 | -10 | *Target* | |
| BUT4 | NADH | -1 | 10 | -10 | *Target* | |
| BUT5 | NADH | -1 | 10 | -10 | *Target* | |
