# Supplementary material for: In silico co-factor balance estimation using constraint-based modelling informs metabolic engineering in Escherichia coli
Source: PLoS Comput Biol. 2020 Aug 10;16(8):e1008125. doi: 10.1371/journal.pcbi.1008125 (PMC7440669; doi:10.1371/journal.pcbi.1008125)
Supplement: S5 Table — Reaction IDs, relevant co-factor and their stoichiometric coefficient, flux value, balance value and assigned balance category are included. (DOCX) [file pcbi.1008125.s005.docx]

| **Table S5 \| CBA parameters and outputs of unconstrained models under anaerobic conditions.** Reaction IDs, relevant co-factor and their stoichiometric coefficient, flux value, balance value and assigned balance category are included. | | | | | | |
| --- | --- | --- | --- | --- | --- | --- |
| **Reaction ID** | **Co-factor** | **Stoich. coefficient** | **Flux Distribution** | **Balance Value** | **Balance Category** |  |
| **Wild Type (WT)** | | | | | | |
| ATPM | ATP | 1­ | 7.6 | -7.6 | *Waste* |  |
| ACKr | ATP | -1 | -8.297 | 8.297 | *Waste* |  |
| Biomass | ATP | -55.703 | 0.241 | -13.415 | *Biomass* |  |
| PYK | ATP | 1 | 8.184 | 8.184 | *Production* |  |
| ATPS4r | ATP | 1 | -5.067 | -5.067 | *Maintenance* |  |
| PGK | ATP | -1 | -19.360 | 19.360 | *Production* |  |
| PFK | ATP | -1 | 9.761 | -9.761 | *Maintenance* |  |
| THD2 | NADPH | 1 | 4.129 | 4.129 | *Production* |  |
| ICDHyr | NADPH | 1 | 0.260 | 0.260 | *Waste* |  |
| THD2 | NADH | -1 | 4.129 | -4.129 | *Maintenance* |  |
| ADHEr | NADH | -2 | 8.042 | -16.085 | *Waste* |  |
| GAPD | NADH | 1 | 19.340 | 19.360 | *Production* |  |
| Biomass | NADH/NADPH | 3.547/-18.225 | 0.241 | -3.535 | *Biomass* |  |
|  | | | | | | |
| **BuOH-0** | | | | | | |
| PFK | ATP | -1 | 10 | -10 | *Maintenance* |  |
| PGK | ATP | -1 | -20 | 20 | *Production* |  |
| PYK | ATP | 1 | 10 | 10 | *Production* |  |
| ATPM | ATP | -1 | 20 | -20 | *Waste* |  |
| GAPD | NADH | 1 | 20 | 20 | *Production* |  |
| BUT2 | NADH | -1 | 10 | -10 | *Target* |  |
| BUT4 | NADH | -1 | 10 | -10 | *Target* |  |
| BUT5 | NADH | -1 | 10 | -10 | *Target* |  |
| BUT6 | NADH | -1 | 10 | -10 | *Target* |  |
| PDH | NADH | 1 | 20 | 20 | *Waste* |  |
|  | | | | | | |
| **BuOH-1** | | | | | | |
| PFK | ATP | -1 | 10 | -10 | *Maintenance* |  |
| PGK | ATP | -1 | -20 | 20 | *Production* |  |
| PYK | ATP | 1 | 10 | 10 | *Production* |  |
| ATPM | ATP | -1 | 10 | -10 | *Waste* |  |
| ACCOAC | ATP | -1 | 10 | -10 | *Target* |  |
| GAPD | NADH | 1 | 20 | 20 | *Production* |  |
| BUT2 | NADH | -1 | 10 | -10 | *Target* |  |
| BUT4 | NADH | -1 | 10 | -10 | *Target* |  |
| BUT5 | NADH | -1 | 10 | -10 | *Target* |  |
| BUT6 | NADH | -1 | 10 | -10 | *Target* |  |
| PDH | NADH | 1 | 20 | 20 | *Waste* |  |
|  |  |  |  |  |  |  |
| **tpcBuOH** |  |  |  |  |  |  |
| PYK | ATP | 1 | 8.756 | 8.756 | *Production* | |
| ATPS4r | ATP | 1 | 7.6 | 7.6 | *Production* | |
| PGK | ATP | -1 | -18.756 | 18.756 | *Production* | |
| CAR | ATP | -1 | 7.467 | -7.467 | *Target* | |
| ATPS4r | ATP | 1 | -0.045 | -0.045 | *Maintenance* | |
| PFK | ATP | -1 | 8.756 | -8.756 | *Maintenance* | |
| ATPM | ATP | -1 | 7.6 | -7.6 | *Waste* | |
| ADK1 | ATP | -1 | 7.467 | -7.467 | *Waste* | |
| ACKr | ATP | -1 | -3.822 | 3.822 | *Waste* | |
| G6PDH2r | NADPH | 1 | 3.733 | 3.733 | *Production* | |
| GAPD | NADH | 1 | 18.756 | 18.756 | *Production* | |
| CAR | NADPH | -1 | 7.467 | -7.467 | *Target* | |
| BUT2 | NADH | -1 | 7.467 | -7.467 | *Target* | |
| BUT4 | NADH | -1 | 7.467 | -7.467 | *Target* | |
| BUT6 | NADH | -1 | 7.467 | -7.467 | *Target* | |
| GND | NADPH | 1 | 3.733 | 3.733 | *Waste* | |
| PDH | NADH | 1 | 3.644 | 3.644 | *Waste* | |
|  |  |  |  |  |  | |
| **BuOH-2** |  |  |  |  |  | |
| PYK | ATP | 1 | 9.520 | 9.520 | *Production* | |
| ATPS4r | ATP | 1 | -3.440 | -3.440 | *Maintenance* | |
| PGK | ATP | -1 | -19.520 | 19.520 | *Production* | |
| CAR | ATP | -1 | 5.6 | -5.6 | *Target* | |
| ACCOAC | ATP | -1 | 5.6 | -5.6 | *Target* | |
| PFK | ATP | -1 | 9.52 | -9.52 | *Maintenance* | |
| ATPM | ATP | -1 | 7.6 | -7.6 | *Waste* | |
| ADK1 | ATP | -1 | 5.6 | -5.6 | *Waste* | |
| ACKr | ATP | -1 | -8.32 | 8.32 | *Waste* | |
| GAPD | NADH | 1 | 19.520 | 19.520 | *Production* | |
| G6PDH2r | NADPH | 1 | 1.440 | 1.440 | *Production* | |
| THD2 | NADPH | 1 | 2.720 | 2.720 | *Production* | |
| CAR | NADPH | -1 | 5.6 | -5.6 | *Target* | |
| BUT2 | NADH | -1 | 5.6 | -5.6 | *Target* | |
| BUT4 | NADH | -1 | 5.6 | -5.6 | *Target* | |
| BUT6 | NADH | -1 | 5.6 | -5.6 | *Target* | |
| THD2 | NADH | -1 | 2.720 | -2.720 | *Maintenance* | |
| GND | NADPH | 1 | 1.439 | 1.439 | *Waste* | |
|  |  |  |  |  |  | |
| **fasBuOH** |  |  |  |  |  | |
| PYK | ATP | 1 | 9.784 | 9.784 | *Production* | |
| ATPS4r | ATP | 1 | -5.614 | -5.614 | *Maintenance* | |
| PGK | ATP | -1 | -19.784 | 19.784 | *Production* | |
| CAR | ATP | -1 | 5.271 | -5.271 | *Target* | |
| ACCOAC | ATP | -1 | 5.271 | -5.271 | *Target* | |
| PFK | ATP | -1 | 9.784 | -9.784 | *Maintenance* | |
| ADK1 | ATP | -1 | 5.271 | -5.271 | *Waste* | |
| ATPM | ATP | -1 | 7.6 | -7.6 | *Waste* | |
| ACKr | ATP | -1 | -9.242 | 9.242 | *Waste* | |
| G6PDH2r | NADPH | 1 | 0.649 | 0.649 | *Production* | |
| GAPD | NADH | 1 | 19.784 | 19.784 | *Production* | |
| THD2 | NADPH | 1 | 9.242 | 9.242 | *Production* | |
| CAR | NADPH | -1 | 5.271 | -5.271 | *Target* | |
| BUT6 | NADH | -1 | 5.271 | -5.271 | *Target* | |
| 30AR40 | NADPH | -1 | 5.271 | -5.271 | *Maintenance* | |
| EAR40x | NADH | -1 | 5.271 | -5.271 | *Maintenance* | |
| THD2 | NADH | -1 | 9.242 | -9.242 | *Maintenance* | |
| GND | NADPH | 1 | 0.649 | 0.649 | *Waste* | |
| **CROT** |  |  |  |  |  | |
| PYK | ATP | 1 | 10 | 10 | *Production* | |
| ATPS4r | ATP | 1 | -3.333 | -3.333 | *Maintenance* | |
| PGK | ATP | -1 | -20 | 20 | *Production* | |
| PFK | ATP | -1 | 10 | -10 | *Maintenance* | |
| ATPM | ATP | -1 | 16.667 | -16.667 | *Waste* | |
| GAPD | NADH | 1 | 20 | 20 | *Production* | |
| BUT2 | NADH | -1 | 6.667 | -6.667 | *Target* | |
| ADHEr | NADH | -1 | 13.333 | -13.333 | *Waste* | |
|  |  |  |  |  |  | |
| **BUTYR** |  |  |  |  |  | |
| PYK | ATP | 1 | 10 | 10 | *Production* | |
| PGK | ATP | -1 | -20 | 20 | *Production* | |
| ATPS4r | ATP | 1 | -5 | -5 | *Maintenance* | |
| PFK | ATP | -1 | 10 | -10 | *Maintenance* | |
| ATPM | ATP | -1 | 15 | -15 | *Waste* | |
| GAPD | NADH | 1 | 20 | 20 | *Production* | |
| BUT2 | NADH | -1 | 10 | -10 | *Target* | |
| BUT4 | NADH | -1 | 10 | -10 | *Target* | |
|  |  |  |  |  |  | |
| **BUTAL** |  |  |  |  |  | |
| PYK | ATP | 1 | 10 | 10 | *Production* | |
| PGK | ATP | -1 | -20 | 20 | *Production* | |
| ATPS4r | ATP | 1 | -2.5 | -2.5 | *Maintenance* | |
| PFK | ATP | -1 | 10 | -10 | *Maintenance* | |
| ATPM | ATP | -1 | 17.5 | -17.5 | *Waste* | |
| GAPD | NADH | 1 | 20 | 20 | *Production* | |
| PDH | NADH | 1 | 10 | 10 | *Waste* | |
| BUT2 | NADH | -1 | 10 | -10 | *Target* | |
| BUT4 | NADH | -1 | 10 | -10 | *Target* | |
| BUT5 | NADH | -1 | 10 | -10 | *Target* | |
