# Supplementary material for: In silico co-factor balance estimation using constraint-based modelling informs metabolic engineering in Escherichia coli
Source: PLoS Comput Biol. 2020 Aug 10;16(8):e1008125. doi: 10.1371/journal.pcbi.1008125 (PMC7440669; doi:10.1371/journal.pcbi.1008125)
Supplement: S6 Table — (DOCX) [file pcbi.1008125.s006.docx]

| **Table S6 \| Candidate reactions for manual curation** | | | | | |  |  |  |  |  |
| --- | --- | --- | --- | --- | --- | --- | --- | --- | --- | --- |
| **Reaction abbreviations** | | | **Reaction name** | **gene** | **Models constrained** | | | | |  |
| **NAD(P)H metabolism** | | | | | | | |  |  |  |
| THD2 | NAD(P) transhydrogenase (periplasm) | | | *pnt* | *tpcBuOH, BuOH-2, fasBuOH, CROT, BUTYR, BUTAL* | | | | | |
| ME1 | malic enzyme (NAD) | | | *sfc* | *BuOH-1, tpcBuOH, BuOH-2, fasBuOH, BUTYR* | | | | | |
| ME2 | malic enzyme (NADP) | | | *mae* | *BuOH-1, tpcBuOH, BuOH-2, fasBuOH, CROT, BUTYR, BUTAL* | | | | | |
| NADTRHD |  | | | *pnt* | *CROT* | | | | | |
| SUCCt2b | succinate efflux via proton import | | | *dcuc* | *tpcBuOH, BuOH-2, fasBuOH* | | | | | |
| PFL | pyruvate formate lyase | | | *pflc* | *tpcBuOH, BuOH-2, fasBuOH* | | | | | |
| **ATP metabolism** | | | | | | | | |  |  |
| ATPM | | ATP maintenance reaction | |  | *BuOH-0, BuOH-1, tpcBuOH, BuOH-2, fasBuOH, CROT, BUTYR, BUTAL* | | | | |  |
| FBP | | fructose bi-phosphate aldolase | | *fbp* | *BuOH-0, BuOH-1, tpcBuOH, BuOH-2, fasBuOH, CROT, BUTYR, BUTAL* | | | | |  |
| ADK1 | | aldenylate kinase | | *adk* | *CROT, BUTYR, BUTAL* | | | | |  |
| PPC | | phosphoenolpyruvate carboxylase | | *ppc* | *BuOH-0, BuOH-1, tpcBuOH, BuOH-2, fasBuOH, CROT, BUTYR, BUTAL* | | | | |  |
| PPS | | phosphoenolpyruvate synthase | | *ppsa* | *BuOH-0, BuOH-1, tpcBuOH, BuOH-2, fasBuOH* | | | | |  |
| ATPS4r | | ATP synthase | | *atpF0, atpF1, atpI* | *BuOH-0, BuOH-1, CROT, BUTYR, BUTAL* | | | | |  |
