# Supplementary material for: In silico co-factor balance estimation using constraint-based modelling informs metabolic engineering in Escherichia coli
Source: PLoS Comput Biol. 2020 Aug 10;16(8):e1008125. doi: 10.1371/journal.pcbi.1008125 (PMC7440669; doi:10.1371/journal.pcbi.1008125)
Supplement: S8 Table — Reaction IDs, relevant co-factor and their stoichiometric coefficient, flux value, balance value and assigned balance category are included. (DOCX) [file pcbi.1008125.s008.docx]

| **Table S8 \| CBA parameters and outputs of manually curated engineered models under anaerobic conditions.** Reaction IDs, relevant co-factor and their stoichiometric coefficient, flux value, balance value and assigned balance category are included. | | | | | | |
| --- | --- | --- | --- | --- | --- | --- |
| **Reaction ID** | **Co-factor** | **Stoich. coefficient** | **Flux Distribution** | **Balance Value** | **Balance Category** |  |
| **BuOH-0** | | | | | | |
| PFK | ATP | -1 | 9.821 | -9.821 | *Maintenance* |  |
| PGK | ATP | -1 | -19.555 | 19.555 | *Production* |  |
| PYK | ATP | 1 | 6.764 | 6.764 | *Production* |  |
| ATPM | ATP | -1 | 7.60 | -7.60 | *Waste* |  |
| Biomass | ATP | -55.703 | 0.160 | -8.899 | *Biomass* |  |
| GAPD | NADH | 1 | 19.555 | 19.555 | *Production* |  |
| G6PDH2r | NADPH | 1 | 0.062 | 0.062 | *Production* |  |
| THD2 | NADPH | 1 | 0.605 | 0.605 | *Production* |  |
| BUT2 | NADH | -1 | 8.776 | -8.776 | *Target* |  |
| BUT4 | NADH | -1 | 8.776 | -8.776 | *Target* |  |
| BUT5 | NADH | -1 | 8.776 | -8.776 | *Target* |  |
| BUT6 | NADH | -1 | 8.776 | -8.776 | *Target* |  |
| MDH | NADH | 1 | -2.011 | -2.011 | *Maintenance* |  |
| NADH11 | NADH | -1 | 0.725 | -0.725 | *Maintenance* |  |
| THD2 | NADH | -1 | 0.605 | -0.605 | *Maintenance* |  |
| PDH | NADH | 1 | 18.323 | 18.323 | *Waste* |  |
| ICDHyr | NADH | 1 | 0.172 | 0.172 | *Waste* |  |
| ME2 | NADH | 1 | 2.011 | 2.011 | *Waste* |  |
| GND | NADH | 1 | 0.062 | 0.062 | *Waste* |  |
| Biomass | NADH/NADPH | 3.547/-18.225 | 0.160 | -2.345 | *Biomass* |  |
|  | | | | | | |
| **BuOH-1** | | | | | | |
| PFK | ATP | -1 | 9.924 | -9.924 | *Maintenance* |  |
| PGK | ATP | -1 | -19.855 | 19.855 | *Production* |  |
| PYK | ATP | 1 | 9.652 | 9.652 | *Production* |  |
| ATPM | ATP | -1 | 7.60 | -7.60 | *Waste* |  |
| ACCOAC | ATP | -1 | 9.667 | -9.667 | *Target* |  |
| Biomass | ATP | -55.703 | 0.045 | -2.316 | *Biomass* |  |
| GAPD | NADH | 1 | 19.855 | 19.855 | *Production* |  |
| G6PDH2r | NADPH | 1 | 0.103 | 0.103 | *Production* |  |
| THD2 | NADPH | 1 | 0.506 | 0.506 | *Production* |  |
| BUT2 | NADH | -1 | 9.667 | -9.667 | *Target* |  |
| BUT4 | NADH | -1 | 9.667 | -9.667 | *Target* |  |
| BUT5 | NADH | -1 | 9.667 | -9.667 | *Target* |  |
| BUT6 | NADH | -1 | 9.667 | -9.667 | *Target* |  |
| NADH11 | NADH | -1 | 0.363 | -0.363 | *Maintenance* |  |
| THD2 | NADH | -1 | 0.506 | -0.506 | *Maintenance* |  |
| ICDHyr | NADH | 1 | 0.049 | 0.049 | *Waste* |  |
| GND | NADPH | 1 | 0.103 | 0.103 | *Waste* |  |
| PDH | NADH | 1 | 19.534 | 19.534 | *Waste* |  |
| Biomass | NADH/NADPH | 3.457/ -18.225 | 0.045 | -0.610 | *Biomass* |  |
|  |  |  |  |  |  |  |
| **tpcBuOH** |  |  |  |  |  |  |
| PYK | ATP | 1 | 7.633 | 7.633 | *Production* | |
| ATPS4r | ATP | 1 | 14.377 | 14.377 | *Production* | |
| PGK | ATP | -1 | -18.082 | 18.082 | *Production* | |
| CAR | ATP | -1 | 8.464 | -8.464 | *Target* | |
| PFK | ATP | -1 | 8.235 | -8.235 | *Maintenance* | |
| ATPM | ATP | -1 | 7.60 | -7.60 | *Waste* | |
| ADK1 | ATP | -1 | 8.464 | -8.464 | *Waste* | |
| PPCK | ATP | -1 | 2.205 | -2.205 | *Waste* | |
| Biomass | ATP | -55.703 | 0.092 | -5.123 | *Biomass* | |
| GAPD | NADH | 1 | 18.082 | 18.082 | *Production* | |
| G6PDH2r | NADPH | 1 | 5.021 | 5.021 | *Production* | |
| CAR | NADPH | -1 | 8.464 | -8.464 | *Target* | |
| BUT2 | NADH | -1 | 8.464 | -8.464 | *Target* | |
| BUT4 | NADH | -1 | 8.464 | -8.464 | *Target* | |
| BUT6 | NADH | -1 | 8.464 | -8.464 | *Target* | |
| GND | NADPH | 1 | 5.021 | 5.021 | *Waste* | |
| PDH | NADH | 1 | 17.373 | 17.373 | *Waste* | |
| ICDHyr | NADH | 1 | 0.099 | 0.099 | *Waste* | |
| NADH11 | NADH | -1 | 10.388 | -10.388 | *Maintenance* | |
| Biomass | NADH/NADPH | 3.457/ -18.225 | 0.092 | -1.350 | *Biomass* | |
|  |  |  |  |  |  | |
| **BuOH-2** |  |  |  |  |  | |
| PYK | ATP | 1 | 8.462 | 8.462 | *Production* | |
| ATPS4r | ATP | 1 | 13.846 | 13.846 | *Production* | |
| PGK | ATP | -1 | -18.462 | 18.462 | *Production* | |
| CAR | ATP | -1 | 9.231 | -9.231 | *Target* | |
| ACCOAC | ATP | -1 | 9.231 | -9.231 | *Target* | |
| PFK | ATP | -1 | 8.462 | -8.462 | *Maintenance* | |
| ATPM | ATP | -1 | 4.615 | -4.615 | *Waste* | |
| ADK1 | ATP | -1 | 9.231 | -9.231 | *Waste* | |
| GAPD | NADH | 1 | 18.462 | 18.462 | *Production* | |
| G6PDH2r | NADPH | 1 | 4.615 | 4.615 | *Production* | |
| CAR | NADPH | -1 | 9.231 | -9.049 | *Target* | |
| BUT2 | NADH | -1 | 9.231 | -9.231 | *Target* | |
| BUT4 | NADH | -1 | 9.231 | -9.231 | *Target* | |
| BUT6 | NADH | -1 | 9.231 | -9.231 | *Target* | |
| NADH11 | NADH | -1 | 9.231 | -9.231 | *Maintenance* | |
| GND | NADPH | 1 | 4.615 | 4.615 | *Waste* | |
| PDH | NADH | 1 | 18.462 | 18.462 | *Waste* | |
|  |  |  |  |  |  | |
| **fasBuOH** |  |  |  |  |  | |
| PYK | ATP | 1 | 6.228 | 6.228 | *Production* | |
| ATPS4r | ATP | 1 | 23.219 | 23.219 | *Production* | |
| PGK | ATP | -1 | -16.799 | 16.799 | *Production* | |
| CAR | ATP | -1 | 7.665 | -7.665 | *Target* | |
| ACCOAC | ATP | -1 | 7.665 | -7.665 | *Target* | |
| PFK | ATP | -1 | 6.994 | -6.994 | *Maintenance* | |
| ADK1 | ATP | -1 | 7.665 | -7.665 | *Waste* | |
| ATPM | ATP | -1 | 7.60 | -7.60 | *Waste* | |
| PPCK | ATP | -1 | 2.133 | -2.133 | *Waste* | |
| Biomass | ATP | -55.703 | 0.117 | -6.523 | *Biomass* | |
| G6PDH2r | NADPH | 1 | 8.669 | 8.669 | *Production* | |
| GAPD | NADH | 1 | 16.799 | 16.799 | *Production* | |
| CAR | NADPH | -1 | 7.665 | -7.665 | *Target* | |
| BUT6 | NADH | -1 | 7.665 | -7.665 | *Target* | |
| 30AR40 | NADPH | -1 | 7.665 | -7.665 | *Maintenance* | |
| EAR40x | NADH | -1 | 7.665 | -7.665 | *Maintenance* | |
| NADH11 | NADH | -1 | 17.779 | -17.779 | *Maintenance* | |
| GND | NADPH | 1 | 8.669 | 8.669 | *Waste* | |
| PDH | NADH | 1 | 15.896 | 15.896 | *Waste* | |
| ICDHyr | NADH | 1 | 0.126 | 0.126 | *Waste* | |
| Biomass | NADH/NADPH | 3.457/ -18.225 | 0.117 | -1.719 | *Biomass* | |
|  |  |  |  |  |  | |
| **CROT** |  |  |  |  |  | |
| PYK | ATP | 1 | 8.410 | 8.410 | *Production* | |
| PGK | ATP | -1 | 19.156 | 19.156 | *Production* | |
| PFK | ATP | -1 | 9.410 | -9.410 | *Maintenance* | |
| ATPM | ATP | -1 | 7.60 | -7.60 | *Waste* | |
| PPCK | ATP | -1 | 2.030 | -2.030 | *Waste* | |
| Biomass | ATP | -55.703 | 0.153 | -8.525 | *Biomass* | |
| GAPD | NADH | 1 | 19.156 | 19.156 | *Production* | |
| G6PDH2r | NADPH | 1 | 1.312 | 1.312 | *Production* | |
| BUT2 | NADH | -1 | 8.618 | -8.618 | *Target* | |
| NADH11 | NADH | -1 | 11.080 | -11.080 | *Maintenance* | |
| ICDHyr | NADH | 1 | 0.165 | 0.165 | *Waste* | |
| GND | NADPH | 1 | 1.312 | 1.312 | *Waste* | |
| Biomass | NADH/NADPH | 3.457/ -18.225 | 0.153 | -2.246 | *Biomass* | |
|  |  |  |  |  |  | |
| **BUTYR** |  |  |  |  |  | |
| PYK | ATP | 1 | 8.409 | 8.409 | *Production* | |
| PGK | ATP | -1 | -19.156 | 19.156 | *Production* | |
| PFK | ATP | -1 | 9.410 | -9.410 | *Maintenance* | |
| ATPM | ATP | -1 | 7.60 | -7.60 | *Waste* | |
| PPCK | ATP | -1 | 2.030 | -2.030 | *Waste* | |
| Biomass | ATP | -55.703 | 0.153 | -8.525 | *Biomass* | |
| GAPD | NADH | 1 | 19.156 | 19.156 | *Production* | |
| G6PDH2r | NADPH | 1 | 1.312 | 1.312 | *Production* | |
| BUT2 | NADH | -1 | 8.618 | -8.618 | *Target* | |
| BUT4 | NADH | -1 | 8.618 | -8.618 | *Target* | |
| NADH11 | NADH | -1 | 4.411 | -4.411 | *Maintenance* | |
| ICDHyr | NADH | 1 | 0.165 | 0.165 | *Waste* | |
| GND | NADPH | 1 | 1.312 | 1.312 | *Waste* | |
| PDH | NADH | 1 | 1.949 | 1.949 | *Waste* | |
| Biomass | NADH/NADPH | 3.457/ -18.225 | 0.153 | -2.246 | *Biomass* | |
|  |  |  |  |  |  | |
| **BUTAL** |  |  |  |  |  | |
| PYK | ATP | 1 | 8.409 | 8.409 | *Production* | |
| PGK | ATP | -1 | -19.156 | 19.156 | *Production* | |
| ATPS4r | ATP | 1 | -2.5 | -2.5 | *Maintenance* | |
| PFK | ATP | -1 | 9.410 | -9.410 | *Maintenance* | |
| ATPM | ATP | -1 | 7.60 | -7.60 | *Waste* | |
| PPCK | ATP | -1 | 2.030 | -2.030 | *Waste* | |
| Biomass | ATP | -55.703 | 0.153 | -8.525 | *Biomass* | |
| GAPD | NADH | 1 | 19.156 | 19.156 | *Production* | |
| G6PDH2r | NADPH | 1 | 1.312 | 1.312 | *Production* | |
| NADH11 | NADH | -1 | 2.688 | -2.688 | *Maintenance* | |
| PDH | NADH | 1 | 8.844 | 8.844 | *Waste* | |
| ICDHyr | NADH | 1 | 0.165 | 0.165 | *Waste* | |
| GND | NADPH | 1 | 1.312 | 1.312 | *Waste* | |
| BUT2 | NADH | -1 | 8.618 | -8.618 | *Target* | |
| BUT4 | NADH | -1 | 8.618 | -8.618 | *Target* | |
| BUT5 | NADH | -1 | 8.618 | -8.618 | *Target* | |
| Biomass | NADH/NADPH | 3.457/ -18.225 | 0.153 | -2.246 | *Biomass* | |
