# Supplementary material for: In silico co-factor balance estimation using constraint-based modelling informs metabolic engineering in Escherichia coli
Source: PLoS Comput Biol. 2020 Aug 10;16(8):e1008125. doi: 10.1371/journal.pcbi.1008125 (PMC7440669; doi:10.1371/journal.pcbi.1008125)
Supplement: S9 Table — Used 100% of optimum and optimized for butanol or butanol precursor production. Minimal and maximal range units are in mmol gDW-1 hr-1. Highlighted in grey–reactions presenting variability ranges, instead of unique fluxes. (DOCX) [file pcbi.1008125.s009.docx]

| Table S9 \| **Flux Variability Analysis of the manually curated engineered models using the *Escherichia coli* Core Model under aerobic conditions.** Used 100% of optimum and optimized for butanol production, accordingly. Minimal and maximal range units are in mmol gDW^-1^ hr^-1^. Highlighted in grey – reactions presenting variability ranges, instead of unique fluxes. | | | | | | | | | | | |
| --- | --- | --- | --- | --- | --- | --- | --- | --- | --- | --- | --- |
| Reaction ID | BuOH-0 | | BuOH-1 | | tpcBuOH | | BuOH-2 | | fasBuOH | |  |
|  | Min | Max | Min | Max | Min | Max | Min | Max | Min | Max |  |
| ACONT | 0.17 | 0.17 | 0.18 | 0.18 | 0.10 | 0.10 | 0.00 | 0.00 | 0.13 | 0.13 |  |
| ADK1 | 0.00 | 0.00 | 0.00 | 0.00 | 8.46 | 8.46 | 9.23 | 9.23 | 7.67 | 7.67 |  |
| ATPM | 7.60 | 7.60 | 7.60 | 7.60 | 7.60 | 7.60 | 2.15 | 4.62 | 7.60 | 7.60 |  |
| ATPS4r | 0.00 | 0.00 | 0.00 | 0.00 | 14.38 | 14.38 | 13.85 | 13.85 | 23.22 | 23.22 |  |
| Biomass | 0.16 | 0.16 | 0.05 | 0.05 | 0.09 | 0.09 | 0.00 | 0.00 | 0.12 | 0.12 |  |
| CS | 0.17 | 0.17 | 0.18 | 0.18 | 0.10 | 0.10 | 0.00 | 0.00 | 0.13 | 0.13 |  |
| CYTBD | 0.73 | 0.73 | 0.65 | 0.95 | 10.39 | 10.39 | 9.23 | 9.23 | 17.78 | 17.78 |  |
| ENO | 19.32 | 19.32 | 19.81 | 19.81 | 17.94 | 17.94 | 18.46 | 18.46 | 16.62 | 16.62 |  |
| FBA | 9.82 | 9.82 | 9.96 | 9.96 | 8.24 | 8.24 | 8.46 | 8.46 | 6.99 | 6.99 |  |
| FRD | 0.00 | 999999 | 0.00 | 999998.8 | 0.00 | 999999 | 0.00 | 999999 | 0.00 | 999999 |  |
| FUM | 0.00 | 0.00 | 0.13 | 0.13 | 0.00 | 0.00 | 0.00 | 0.00 | 0.00 | 0.00 |  |
| G6PDH2r | 0.06 | 0.06 | 0.00 | 0.00 | 5.02 | 5.02 | 4.62 | 4.62 | 8.67 | 8.67 |  |
| GAPD | 19.56 | 19.56 | 19.88 | 19.88 | 18.08 | 18.08 | 18.46 | 18.46 | 16.80 | 16.80 |  |
| GLCpts | 10.00 | 10.00 | 10.00 | 10.00 | 10.00 | 10.00 | 10.00 | 10.00 | 10.00 | 10.00 |  |
| GND | 0.06 | 0.06 | 0.00 | 0.00 | 5.02 | 5.02 | 4.62 | 4.62 | 8.67 | 8.67 |  |
| ICDHyr | 0.17 | 0.17 | 0.05 | 0.05 | 0.10 | 0.10 | 0.00 | 0.00 | 0.13 | 0.13 |  |
| ICL | 0.00 | 0.00 | 0.13 | 0.13 | 0.00 | 0.00 | 0.00 | 0.00 | 0.00 | 0.00 |  |
| MALS | 0.00 | 0.00 | 0.13 | 0.13 | 0.00 | 0.00 | 0.00 | 0.00 | 0.00 | 0.00 |  |
| MDH | -2.01 | -2.01 | 0.26 | 0.26 | 0.00 | 0.00 | 0.00 | 0.00 | 0.00 | 0.00 |  |
| ME1 | 0.00 | 0.00 | 0.00 | 0.00 | 0.00 | 0.00 | 0.00 | 0.00 | 0.00 | 0.00 |  |
| NADH11 | 0.73 | 0.73 | 0.52 | 0.82 | 10.39 | 10.39 | 9.23 | 9.23 | 17.78 | 17.78 |  |
| NADTRHD | 0.00 | 0.00 | 0.00 | 0.75 | 0.00 | 0.00 | 0.00 | 0.00 | 0.00 | 0.00 |  |
| PDH | 18.32 | 18.32 | 19.36 | 19.66 | 17.37 | 17.37 | 18.46 | 18.46 | 15.90 | 15.90 |  |
| PFK | 9.82 | 9.82 | 9.96 | 9.96 | 8.24 | 8.24 | 8.46 | 8.46 | 6.99 | 6.99 |  |
| PFL | 0.00 | 0.00 | 0.00 | 0.30 | 0.00 | 0.00 | 0.00 | 0.00 | 0.00 | 0.00 |  |
| PGI | 9.91 | 9.91 | 9.99 | 9.99 | 4.96 | 4.96 | 5.39 | 5.39 | 1.31 | 1.31 |  |
| PGK | -19.56 | -19.56 | -19.88 | -19.88 | -18.08 | -18.08 | -18.46 | -18.46 | -16.80 | -16.80 |  |
| PGL | 0.06 | 0.06 | 0.00 | 0.00 | 5.02 | 5.02 | 4.62 | 4.62 | 8.67 | 8.67 |  |
| PGM | -19.32 | -19.32 | -19.81 | -19.81 | -17.94 | -17.94 | -18.46 | -18.46 | -16.62 | -16.62 |  |
| PPC | 2.47 | 2.47 | 0.00 | 0.00 | 2.47 | 2.47 | 0.00 | 2.47 | 2.47 | 2.47 |  |
| PPCK | 0.00 | 0.00 | 0.00 | 0.00 | 2.21 | 2.21 | 0.00 | 2.47 | 2.13 | 2.13 |  |
| PYK | 6.76 | 6.76 | 9.79 | 9.79 | 7.63 | 7.63 | 8.46 | 8.46 | 6.23 | 6.23 |  |
| RPE | -0.07 | -0.07 | -0.03 | -0.03 | 3.28 | 3.28 | 3.08 | 3.08 | 5.70 | 5.70 |  |
| RPI | -0.14 | -0.14 | -0.03 | -0.03 | -1.74 | -1.74 | -1.54 | -1.54 | -2.97 | -2.97 |  |
| 22_2 | 0.00 | 0.00 | 0.00 | 1.51 | 0.00 | 0.00 | 0.00 | 0.00 | 0.00 | 0.00 |  |
| SUCCt2b | 0.00 | 0.00 | 0.00 | 1.51 | 0.00 | 0.00 | 0.00 | 0.00 | 0.00 | 0.00 |  |
| SUCD1i | 0.00 | 999999 | 0.13 | 999999 | 0.00 | 999999 | 0.00 | 999999 | 0.00 | 999999 |  |
| SUCD4 | 0.00 | 0.00 | 0.13 | 0.13 | 0.00 | 0.00 | 0.00 | 0.00 | 0.00 | 0.00 |  |
| TALA | -0.01 | -0.01 | -0.01 | -0.01 | 1.66 | 1.66 | 1.54 | 1.54 | 2.87 | 2.87 |  |
| THD2 | 0.61 | 0.61 | 0.78 | 1.53 | 0.00 | 0.00 | 0.00 | 0.00 | 0.00 | 0.00 |  |
| TKT1 | -0.01 | -0.01 | -0.01 | -0.01 | 1.66 | 1.66 | 1.54 | 1.54 | 2.87 | 2.87 |  |
| TKT2 | -0.07 | -0.07 | -0.02 | -0.02 | 1.62 | 1.62 | 1.54 | 1.54 | 2.83 | 2.83 |  |
| TPI | 9.82 | 9.82 | 9.96 | 9.96 | 8.24 | 8.24 | 8.46 | 8.46 | 6.99 | 6.99 |  |
| BUT1 | 8.78 | 8.78 |  |  | 8.46 | 8.46 |  |  |  |  |  |
| BUT2 | 8.78 | 8.78 | 9.59 | 9.59 | 8.46 | 8.46 | 9.23 | 9.23 |  |  |  |
| BUT3 | 8.78 | 8.78 | 9.59 | 9.59 | 8.46 | 8.46 | 9.23 | 9.23 |  |  |  |
| BUT4 | 8.78 | 8.78 | 9.59 | 9.59 | 8.46 | 8.46 | 9.23 | 9.23 |  |  |  |
| BUT5 | 8.78 | 8.78 | 9.59 | 9.59 |  |  |  |  |  |  |  |
| BUT6 | 8.78 | 8.78 | 9.59 | 9.59 | 8.46 | 8.46 | 9.23 | 9.23 | 7.67 | 7.67 |  |
| BTOH_tr | 8.78 | 8.78 | 9.59 | 9.59 | 8.46 | 8.46 | 9.23 | 9.23 | 7.67 | 7.67 |  |
| BTOH_sink | 8.78 | 8.78 | 9.59 | 9.59 | 8.46 | 8.46 | 9.23 | 9.23 | 7.67 | 7.67 |  |
| HCO3E |  |  | 9.59 | 9.59 |  |  | 9.23 | 9.23 | 7.67 | 7.67 |  |
| ACCOAC |  |  | 9.59 | 9.59 |  |  | 9.23 | 9.23 | 7.67 | 7.67 |  |
| NPHT7 |  |  | 9.59 | 9.59 |  |  | 9.23 | 9.23 |  |  |  |
| BTBTAC |  |  |  |  | 8.46 | 8.46 | 9.23 | 9.23 |  |  |  |
| CAR |  |  |  |  | 8.46 | 8.46 | 9.23 | 9.23 | 7.67 | 7.67 |  |
| ACPS1 |  |  |  |  |  |  |  |  | 0.00 | 0.00 |  |
| BPNT |  |  |  |  |  |  |  |  | 0.00 | 0.00 |  |
| MCOATA |  |  |  |  |  |  |  |  | 7.67 | 7.67 |  |
| KAS15 |  |  |  |  |  |  |  |  | 7.67 | 7.67 |  |
| 3OAR40 |  |  |  |  |  |  |  |  | 7.67 | 7.67 |  |
| 3HAD40 |  |  |  |  |  |  |  |  | 7.67 | 7.67 |  |
| EAR40x |  |  |  |  |  |  |  |  | 7.67 | 7.67 |  |
| 5_BUT1 |  |  |  |  |  |  |  |  | 7.67 | 7.67 |  |
| BTBTAC | 0.17 | 0.17 | 0.18 | 0.18 | 0.10 | 0.10 | 0.00 | 0.00 | 0.13 | 0.13 |  |
| BTAC_tr | 0.00 | 0.00 | 0.00 | 0.00 | 8.46 | 8.46 | 9.23 | 9.23 | 7.67 | 7.67 |  |
| BTAC_sink | 7.60 | 7.60 | 7.60 | 7.60 | 7.60 | 7.60 | 2.15 | 4.62 | 7.60 | 7.60 |  |
| BTAL_tr | 0.00 | 0.00 | 0.00 | 0.00 | 14.38 | 14.38 | 13.85 | 13.85 | 23.22 | 23.22 |  |
| BTAL_sink | 0.16 | 0.16 | 0.05 | 0.05 | 0.09 | 0.09 | 0.00 | 0.00 | 0.12 | 0.12 |  |

| Table S9 (continued) \| **Flux Variability Analysis of the manually curated butanol precursor models using the *Escherichia coli* Core Model under aerobic conditions.** Used 100% of optimum and optimized for butanol precursor production, accordingly. Minimal and maximal range units are in mmol gDW^-1^ hr^-1^. Highlighted in grey – reactions presenting variability ranges, instead of unique fluxes. | | | | | | |
| --- | --- | --- | --- | --- | --- | --- |
| Reaction ID | CROT | | BUTYR | | Butal | |
|  | Min | Max | Min | Max | Min | Max |
| ACONT | 0.165 | 0.165 | 0.165 | 0.165 | 0.165 | 0.165 |
| ADK1 | 0 | 0 | 0 | 0 | 0 | 0 |
| ATPM | 7.6 | 7.6 | 7.6 | 7.6 | 7.6 | 7.6 |
| ATPS4r | 0 | 0 | 0 | 0 | 0 | 0 |
| Biomass | 0.153 | 0.153 | 0.153 | 0.153 | 0.153 | 0.153 |
| CS | 0.165 | 0.165 | 0.165 | 0.165 | 0.165 | 0.165 |
| CYTBD | 11.08 | 29.056 | 4.411 | 20.437 | 2.688 | 11.819 |
| ENO | 18.927 | 18.927 | 18.927 | 18.927 | 18.927 | 18.927 |
| FBA | 9.41 | 9.41 | 9.41 | 9.41 | 9.41 | 9.41 |
| FRD | 0 | 999999 | 0 | 999999 | 0 | 999999 |
| FUM | 0 | 0 | 0 | 0 | 0 | 0 |
| G6PDH2r | 1.312 | 1.312 | 1.312 | 1.312 | 1.312 | 1.312 |
| GAPD | 19.156 | 19.156 | 19.156 | 19.156 | 19.156 | 19.156 |
| GLCpts | 10 | 10 | 10 | 10 | 10 | 10 |
| GND | 1.312 | 1.312 | 1.312 | 1.312 | 1.312 | 1.312 |
| ICDHyr | 0.165 | 0.165 | 0.165 | 0.165 | 0.165 | 0.165 |
| ICL | 0 | 0 | 0 | 0 | 0 | 0 |
| MALS | 0 | 0 | 0 | 0 | 0 | 0 |
| MDH | -2.03 | 0 | 0 | 0 | -2.03 | 0 |
| ME1 | 0 | 2.03 | 0 | 0 | 0 | 2.03 |
| NADH11 | 11.08 | 29.056 | 4.411 | 20.437 | 2.688 | 11.819 |
| NADTRHD | 0 | 0 | 0 | 0 | 0 | 0 |
| PDH | 0 | 17.975 | 1.949 | 17.975 | 8.844 | 17.975 |
| PFK | 9.41 | 9.41 | 9.41 | 9.41 | 9.41 | 9.41 |
| PFL | 0 | 17.975 | 0 | 16.026 | 0 | 9.131 |
| PGI | 8.657 | 8.657 | 8.657 | 8.657 | 8.657 | 8.657 |
| PGK | -19.156 | -19.156 | -19.156 | -19.156 | -19.156 | -19.156 |
| PGL | 1.312 | 1.312 | 1.312 | 1.312 | 1.312 | 1.312 |
| PGM | -18.927 | -18.927 | -18.927 | -18.927 | -18.927 | -18.927 |
| PPC | 2.468 | 2.468 | 2.468 | 2.468 | 2.468 | 2.468 |
| PPCK | 0 | 2.03 | 2.03 | 2.03 | 0 | 2.03 |
| PYK | 6.379 | 8.409 | 8.409 | 8.409 | 6.379 | 8.409 |
| RPE | 0.765 | 0.765 | 0.765 | 0.765 | 0.765 | 0.765 |
| RPI | -0.547 | -0.547 | -0.547 | -0.547 | -0.547 | -0.547 |
| 22_2 | 24.727 | 114.604 | 0 | 80.13 | 0 | 45.657 |
| SUCCt2b | 24.727 | 114.604 | 0 | 80.13 | 0 | 45.657 |
| SUCD1i | 0 | 999999 | 0 | 999999 | 0 | 999999 |
| SUCD4 | 0 | 0 | 0 | 0 | 0 | 0 |
| TALA | 0.41 | 0.41 | 0.41 | 0.41 | 0.41 | 0.41 |
| THD2 | 0 | 0 | 0 | 0 | 0 | 0 |
| TKT1 | 0.41 | 0.41 | 0.41 | 0.41 | 0.41 | 0.41 |
| TKT2 | 0.355 | 0.355 | 0.355 | 0.355 | 0.355 | 0.355 |
| TPI | 9.41 | 9.41 | 9.41 | 9.41 | 9.41 | 9.41 |
| BUT1 | 8.618 | 8.618 | 8.618 | 8.618 | 8.618 | 8.618 |
| BUT2 | 8.618 | 8.618 | 8.618 | 8.618 | 8.618 | 8.618 |
| BUT3 | 8.618 | 8.618 | 8.618 | 8.618 | 8.618 | 8.618 |
| BUT4 |  |  | 8.618 | 8.618 | 8.618 | 8.618 |
| BUT5 |  |  |  |  | 8.618 | 8.618 |
| B2CTCRO | 8.618 | 8.618 |  |  |  |  |
| CROAC_tr | 8.618 | 8.618 |  |  |  |  |
| CROT_sink | 8.618 | 8.618 |  |  |  |  |
| BTBTAC |  |  | 8.618 | 8.618 |  |  |
| BTAC_tr |  |  | 8.618 | 8.618 |  |  |
| BTAC_sink |  |  | 8.618 | 8.618 |  |  |
| BTAL_tr |  |  |  |  | 8.618 | 8.618 |
| BTAL_sink |  |  |  |  | 8.618 | 8.618 |
