# Supplementary material for: In silico co-factor balance estimation using constraint-based modelling informs metabolic engineering in Escherichia coli
Source: PLoS Comput Biol. 2020 Aug 10;16(8):e1008125. doi: 10.1371/journal.pcbi.1008125 (PMC7440669; doi:10.1371/journal.pcbi.1008125)
Supplement: S10 Table — Used 100% of optimum and optimized for butanol or butanol precursor production. Minimal and maximal range units are in mmol gDW-1 hr-1. Highlighted in grey–reactions presenting variability ranges, instead of unique fluxes. (DOCX) [file pcbi.1008125.s010.docx]

| Table S10 \| **Flux Variability Analysis of the manually curated butanol models using the *Escherichia coli* Core Model under anaerobic conditions.** Used 100% of optimum and optimized for butanol production, accordingly. Minimal and maximal range units are in mmol gDW^-1^ hr^-1^. Highlighted in grey – reactions presenting variability ranges, instead of unique fluxes. | | | | | | | | | | | |
| --- | --- | --- | --- | --- | --- | --- | --- | --- | --- | --- | --- |
| Reaction ID | BuOH-0 | | BuOH-1 | | tpcBuOH | | BuOH-2 | | fasBuOH | |  |
|  | Min | Max | Min | Max | Min | Max | Min | Max | Min | Max |  |
| ACKr | 0.00 | 0.00 | 0.00 | 0.00 | 0.00 | 0.00 | -3.75 | -3.75 | -4.18 | -4.18 |  |
| ACONT | 0.20 | 0.20 | 0.05 | 0.05 | 0.00 | 0.00 | 0.00 | 0.00 | 0.00 | 0.00 |  |
| ACt2r | 0.00 | 0.00 | 0.00 | 0.00 | 0.00 | 0.00 | -3.75 | -3.75 | -4.18 | -4.18 |  |
| ADHEr | 5.53 | 5.53 | 1.34 | 1.34 | 0.00 | 0.00 | 0.00 | 0.00 | 0.05 | 0.05 |  |
| ADK1 | 0.00 | 0.00 | 0.00 | 0.00 | 9.55 | 9.55 | 7.50 | 7.50 | 7.06 | 7.06 |  |
| ATPM | 7.60 | 7.60 | 7.60 | 7.60 | 0.00 | 0.00 | 0.00 | 0.00 | 0.00 | 0.00 |  |
| ATPS4r | 0.00 | 0.00 | 0.00 | 0.00 | 0.00 | 0.00 | 0.00 | 0.00 | -1.35 | -1.35 |  |
| Biomass | 0.18 | 0.18 | 0.04 | 0.04 | 0.00 | 0.00 | 0.00 | 0.00 | 0.00 | 0.00 |  |
| CS | 0.20 | 0.20 | 0.05 | 0.05 | 0.00 | 0.00 | 0.00 | 0.00 | 0.00 | 0.00 |  |
| ENO | 18.76 | 18.76 | 19.70 | 19.70 | 19.10 | 19.10 | 18.75 | 18.75 | 18.34 | 18.34 |  |
| ETOHt2r | -5.53 | -5.53 | -1.34 | -1.34 | 0.00 | 0.00 | 0.00 | 0.00 | -0.05 | -0.05 |  |
| FBA | 9.33 | 9.33 | 9.84 | 9.84 | 9.10 | 9.10 | 8.75 | 8.75 | 8.34 | 8.34 |  |
| FORt | -3.61 | -3.61 | -0.89 | -0.89 | -5.42 | -5.42 | -15.00 | -15.00 | -18.34 | -18.34 |  |
| FRD | 0.00 | 999999 | 0.00 | 999999.00 | 0.00 | 999999 | 0.00 | 999999 | 0.00 | 999999 |  |
| G6PDH2r | 1.47 | 1.47 | 0.37 | 0.37 | 2.71 | 2.71 | 3.75 | 3.75 | 4.99 | 4.99 |  |
| GAPD | 19.03 | 19.03 | 19.77 | 19.77 | 19.10 | 19.10 | 18.75 | 18.75 | 18.34 | 18.34 |  |
| GLCpts | 10.00 | 10.00 | 10.00 | 10.00 | 10.00 | 10.00 | 10.00 | 10.00 | 10.00 | 10.00 |  |
| GND | 1.47 | 1.47 | 0.37 | 0.37 | 2.71 | 2.71 | 3.75 | 3.75 | 4.99 | 4.99 |  |
| H2Ot | -4.35 | -4.35 | -8.63 | -8.63 | -25.94 | -25.94 | -15.00 | -15.00 | -12.00 | -12.00 |  |
| ICDHyr | 0.20 | 0.20 | 0.05 | 0.05 | 0.00 | 0.00 | 0.00 | 0.00 | 0.00 | 0.00 |  |
| MDH | -0.17 | -0.17 | 0.00 | 0.00 | 0.00 | 0.00 | 0.00 | 0.00 | 0.00 | 0.00 |  |
| ME2 | 0.17 | 0.17 | 0.00 | 0.00 | 0.00 | 0.00 | 0.00 | 0.00 | 0.00 | 0.00 |  |
| PDH | 14.03 | 14.03 | 18.55 | 18.55 | 13.68 | 13.68 | 3.75 | 3.75 | 0.00 | 0.00 |  |
| PFK | 9.33 | 9.33 | 9.84 | 9.84 | 9.10 | 9.10 | 8.75 | 8.75 | 8.34 | 8.34 |  |
| PFL | 3.61 | 3.61 | 0.89 | 0.89 | 5.42 | 5.42 | 15.00 | 15.00 | 18.34 | 18.34 |  |
| PGI | 8.50 | 8.50 | 9.63 | 9.63 | 7.29 | 7.29 | 6.25 | 6.25 | 5.01 | 5.01 |  |
| PGK | -19.03 | -19.03 | -19.77 | -19.77 | -19.10 | -19.10 | -18.75 | -18.75 | -18.34 | -18.34 |  |
| PGL | 1.47 | 1.47 | 0.37 | 0.37 | 2.71 | 2.71 | 3.75 | 3.75 | 4.99 | 4.99 |  |
| PGM | -18.76 | -18.76 | -19.70 | -19.70 | -19.10 | -19.10 | -18.75 | -18.75 | -18.34 | -18.34 |  |
| PIt | -0.67 | -0.67 | -0.16 | -0.16 | 0.00 | 0.00 | 0.00 | 0.00 | 0.00 | 0.00 |  |
| PPC | 0.69 | 0.69 | 0.69 | 0.69 | 0.00 | 0.00 | 0.00 | 0.00 | 0.00 | 0.00 |  |
| PPCK | 0.00 | 0.00 | 0.57 | 0.57 | 0.00 | 0.00 | 0.00 | 0.00 | 0.00 | 0.00 |  |
| PTAr | 0.00 | 0.00 | 0.00 | 0.00 | 0.00 | 0.00 | 3.75 | 3.75 | 4.18 | 4.18 |  |
| PYK | 7.98 | 7.98 | 9.56 | 9.56 | 9.10 | 9.10 | 8.75 | 8.75 | 8.34 | 8.34 |  |
| RPE | 0.85 | 0.85 | 0.21 | 0.21 | 1.81 | 1.81 | 2.50 | 2.50 | 3.33 | 3.33 |  |
| RPI | -0.62 | -0.62 | -0.15 | -0.15 | -0.90 | -0.90 | -1.25 | -1.25 | -1.66 | -1.66 |  |
| 22_2 | 0.00 | 0.00 | 0.00 | 0.00 | 5.42 | 5.42 | 0.00 | 0.00 | 0.00 | 0.00 |  |
| SUCCt2b | 0.00 | 0.00 | 0.00 | 0.00 | 5.42 | 5.42 | 0.00 | 0.00 | 0.00 | 0.00 |  |
| SUCD1i | 0.00 | 999999 | 0.00 | 999999.00 | 0.00 | 999999 | 0.00 | 999999 | 0.00 | 999999 |  |
| TALA | 0.46 | 0.46 | 0.11 | 0.11 | 0.90 | 0.90 | 1.25 | 1.25 | 1.66 | 1.66 |  |
| THD2 | 0.00 | 0.00 | 0.00 | 0.00 | 4.13 | 4.13 | 0.00 | 0.00 | 4.13 | 4.13 |  |
| TKT1 | 0.46 | 0.46 | 0.11 | 0.11 | 0.90 | 0.90 | 1.25 | 1.25 | 1.66 | 1.66 |  |
| TKT2 | 0.39 | 0.39 | 0.10 | 0.10 | 0.90 | 0.90 | 1.25 | 1.25 | 1.66 | 1.66 |  |
| TPI | 9.33 | 9.33 | 9.84 | 9.84 | 9.10 | 9.10 | 8.75 | 8.75 | 8.34 | 8.34 |  |
| BUT1 | 5.62 | 5.62 |  |  | 9.55 | 9.55 |  |  |  |  |  |
| BUT2 | 5.62 | 5.62 | 8.94 | 8.94 | 9.55 | 9.55 | 7.50 | 7.50 |  |  |  |
| BUT3 | 5.62 | 5.62 | 8.94 | 8.94 | 9.55 | 9.55 | 7.50 | 7.50 |  |  |  |
| BUT4 | 5.62 | 5.62 | 8.94 | 8.94 | 9.55 | 9.55 | 7.50 | 7.50 |  |  |  |
| BUT5 | 5.62 | 5.62 | 8.94 | 8.94 |  |  |  |  |  |  |  |
| BUT6 | 5.62 | 5.62 | 8.94 | 8.94 | 9.55 | 9.55 | 7.50 | 7.50 | 7.06 | 7.06 |  |
| BTOH_tr | 5.62 | 5.62 | 8.94 | 8.94 | 9.55 | 9.55 | 7.50 | 7.50 | 7.06 | 7.06 |  |
| BTOH_sink | 5.62 | 5.61 | 8.94 | 8.94 | 9.55 | 9.55 | 7.50 | 7.50 | 7.06 | 7.06 |  |
| HCO3E |  |  | 8.94 | 8.94 |  |  | 7.50 | 7.50 | 7.06 | 7.06 |  |
| ACCOAC |  |  | 8.94 | 8.94 |  |  | 7.50 | 7.50 | 7.06 | 7.06 |  |
| NPHT7 |  |  | 8.94 | 8.94 |  |  | 7.50 | 7.50 | 7.06 | 7.06 |  |
| BTBTAC |  |  |  |  | 9.55 | 9.55 | 7.50 | 7.50 |  |  |  |
| CAR |  |  |  |  | 9.55 | 9.55 | 7.50 | 7.50 |  |  |  |
| MCOATA |  |  |  |  |  |  |  |  | 7.06 | 7.06 |  |
| KAS15 |  |  |  |  |  |  |  |  | 7.06 | 7.06 |  |
| 3OAR40 |  |  |  |  |  |  |  |  | 7.06 | 7.06 |  |
| 3HAD40 |  |  |  |  |  |  |  |  | 7.06 | 7.06 |  |
| EAR40x |  |  |  |  |  |  |  |  | 7.06 | 7.06 |  |
| 5_BUT1 |  |  |  |  |  |  |  |  | 7.06 | 7.06 |  |
| ACKr | 0.00 | 0.00 | 0.00 | 0.00 | 0.00 | 0.00 | -3.75 | -3.75 | -4.18 | -4.18 |  |
| ACONT | 0.20 | 0.20 | 0.05 | 0.05 | 0.00 | 0.00 | 0.00 | 0.00 | 0.00 | 0.00 |  |
| ACt2r | 0.00 | 0.00 | 0.00 | 0.00 | 0.00 | 0.00 | -3.75 | -3.75 | -4.18 | -4.18 |  |
| ADHEr | 5.53 | 5.53 | 1.34 | 1.34 | 0.00 | 0.00 | 0.00 | 0.00 | 0.05 | 0.05 |  |
| ADK1 | 0.00 | 0.00 | 0.00 | 0.00 | 9.55 | 9.55 | 7.50 | 7.50 | 7.06 | 7.06 |  |
| ATPM | 7.60 | 7.60 | 7.60 | 7.60 | 0.00 | 0.00 | 0.00 | 0.00 | 0.00 | 0.00 |  |

| Table S10 (continued) \| **Flux Variability Analysis of the manually curated butanol precursor models using the *Escherichia coli* Core Model under anaerobic conditions.** Used 100% of optimum and optimized for butanol precursor production, accordingly. Minimal and maximal range units are in mmol gDW^-1^ hr^-1^. Highlighted in grey – reactions presenting variability ranges, instead of unique fluxes. | | | | | | | | |  |  |
| --- | --- | --- | --- | --- | --- | --- | --- | --- | --- | --- |
| Reaction ID | CROT | | BUTYR | | | Butal | | | | |
|  | Min | Max | | Min | Max | | Min | Max | |  |
| ACKr | 0 | 0 | | 0 | 0 | | 0 | 0 | |  |
| ACONT | 0.195 | 0.195 | | 0.195 | 0.195 | | 0.195 | 0.195 | |  |
| ACt2r | 0 | 0 | | 0 | 0 | | 0 | 0 | |  |
| ADHEr | 12.318 | 12.318 | | 11.212 | 11.212 | | 9.369 | 9.369 | |  |
| ADK1 | 0 | 0 | | 0 | 0 | | 0 | 0 | |  |
| ATPM | 7.6 | 7.6 | | 7.6 | 7.6 | | 7.6 | 7.6 | |  |
| ATPS4r | 0 | 0 | | 0 | 0 | | 0 | 0 | |  |
| Biomass | 0.18 | 0.18 | | 0.18 | 0.18 | | 0.18 | 0.18 | |  |
| CS | 0.195 | 0.195 | | 0.195 | 0.195 | | 0.195 | 0.195 | |  |
| ENO | 18.735 | 18.735 | | 18.735 | 18.735 | | 18.735 | 18.735 | |  |
| ETOHt2r | -12.318 | -12.318 | | -11.212 | -11.212 | | -9.369 | -9.369 | |  |
| FBA | 9.305 | 9.305 | | 9.305 | 9.305 | | 9.305 | 9.305 | |  |
| FORt | -10.409 | -10.409 | | -9.303 | -9.303 | | -7.46 | -7.46 | |  |
| FRD | 0 | 999999 | | 0 | 999999 | | 0 | 999999 | |  |
| G6PDH2r | 1.547 | 1.547 | | 1.547 | 1.547 | | 1.547 | 1.547 | |  |
| GAPD | 19.005 | 19.005 | | 19.005 | 19.005 | | 19.005 | 19.005 | |  |
| GLCpts | 10 | 10 | | 10 | 10 | | 10 | 10 | |  |
| GND | 1.547 | 1.547 | | 1.547 | 1.547 | | 1.547 | 1.547 | |  |
| H2Ot | 1.348 | 1.348 | | 1.348 | 1.348 | | -2.339 | -2.339 | |  |
| ICDHyr | 0.195 | 0.195 | | 0.195 | 0.195 | | 0.195 | 0.195 | |  |
| MDH | 0 | 0 | | 0 | 0 | | 0 | 0 | |  |
| ME2 | 0 | 0 | | 0 | 0 | | 0 | 0 | |  |
| PDH | 7.204 | 7.204 | | 8.31 | 8.31 | | 10.153 | 10.153 | |  |
| PFK | 9.305 | 9.305 | | 9.305 | 9.305 | | 9.305 | 9.305 | |  |
| PFL | 10.409 | 10.409 | | 9.303 | 9.303 | | 7.46 | 7.46 | |  |
| PGI | 8.416 | 8.416 | | 8.416 | 8.416 | | 8.416 | 8.416 | |  |
| PGK | -19.005 | -19.005 | | -19.005 | -19.005 | | -19.005 | -19.005 | |  |
| PGL | 1.547 | 1.547 | | 1.547 | 1.547 | | 1.547 | 1.547 | |  |
| PGM | -18.735 | -18.735 | | -18.735 | -18.735 | | -18.735 | -18.735 | |  |
| PIt | -0.664 | -0.664 | | -0.664 | -0.664 | | -0.664 | -0.664 | |  |
| PPC | 0.69 | 0.69 | | 0.69 | 0.69 | | 0.69 | 0.69 | |  |
| PPCK | 0.173 | 0.173 | | 0.173 | 0.173 | | 0.173 | 0.173 | |  |
| PTAr | 0 | 0 | | 0 | 0 | | 0 | 0 | |  |
| PYK | 8.124 | 8.124 | | 8.124 | 8.124 | | 8.124 | 8.124 | |  |
| RPE | 0.902 | 0.902 | | 0.902 | 0.902 | | 0.902 | 0.902 | |  |
| RPI | -0.645 | -0.645 | | -0.645 | -0.645 | | -0.645 | -0.645 | |  |
| 22_2 | 0 | 0 | | 0 | 0 | | 0 | 0 | |  |
| SUCCt2b | 0 | 0 | | 0 | 0 | | 0 | 0 | |  |
| SUCD1i | 0 | 999999 | | 0 | 999999 | | 0 | 999999 | |  |
| TALA | 0.483 | 0.483 | | 0.483 | 0.483 | | 0.483 | 0.483 | |  |
| THD2 | 0 | 0 | | 0 | 0 | | 0 | 0 | |  |
| TKT1 | 0.483 | 0.483 | | 0.483 | 0.483 | | 0.483 | 0.483 | |  |
| TKT2 | 0.418 | 0.418 | | 0.418 | 0.418 | | 0.418 | 0.418 | |  |
| TPI | 9.305 | 9.305 | | 9.305 | 9.305 | | 9.305 | 9.305 | |  |
| BUT1 | 2.212 | 2.212 | | 2.765 | 2.765 | | 3.687 | 3.687 | |  |
| BUT2 | 2.212 | 2.212 | | 2.765 | 2.765 | | 3.687 | 3.687 | |  |
| BUT3 | 2.212 | 2.212 | | 2.765 | 2.765 | | 3.687 | 3.687 | |  |
| BUT4 |  |  | | 2.765 | 2.765 | | 3.687 | 3.687 | |  |
| BUT5 |  |  | |  |  | | 3.687 | 3.687 | |  |
| B2CTCRO | 2.212 | 2.212 | |  |  | |  |  | |  |
| CROAC_tr | 2.212 | 2.212 | |  |  | |  |  | |  |
| CROT_sink | 2.212 | 2.212 | |  |  | |  |  | |  |
| BTBTAC |  |  | | 2.765 | 2.765 | |  |  | |  |
| BTAC_tr |  |  | | 2.765 | 2.765 | |  |  | |  |
| BTAC_sink |  |  | | 2.765 | 2.765 | |  |  | |  |
| BTAL_tr |  |  | |  |  | | 3.687 | 3.687 | |  |
| BTAL_sink |  |  | |  |  | | 3.687 | 3.687 | |  |
