# Supplementary material for: In silico co-factor balance estimation using constraint-based modelling informs metabolic engineering in Escherichia coli
Source: PLoS Comput Biol. 2020 Aug 10;16(8):e1008125. doi: 10.1371/journal.pcbi.1008125 (PMC7440669; doi:10.1371/journal.pcbi.1008125)
Supplement: S11 Table — Reaction-specific stoichiometric coefficients were calculated per reaction involved in the synthetic pathway, and the final pathway coefficient was retrieved as the net sum across all reactions. Respiro-fermentative conditions assumed that the release of 2 mol of acetyl-CoA yields 2 mol ATP, 2 CO2 and 4 mol NADH per mol of carbon source assimilated (glucose, C6H12O6) prior to product formation. This was accounted for in the below calculations. (DOCX) [file pcbi.1008125.s011.docx]

| **Table S11 \| Pathway coefficients for NADPH demand, product release, ATP release, NADH release and CO2 release of all butanol and butanol precursor pathways.** Reaction-specific stoichiometric coefficients were calculated per reaction involved in the synthetic pathway, and the final pathway coefficient was retrieved as the net sum across all reactions. Respiro-fermentative conditions assumed that the release of 2 mol of acetyl-CoA yields 2 mol ATP, 2 CO_2_ and 4 mol NADH per mol of carbon source assimilated (glucose, C_6_H_12_O_6_) prior to product formation. This was accounted for in the below calculations. | | | | | | |
| --- | --- | --- | --- | --- | --- | --- |
| Pathway | Product | a (NADPH) | b (product) | c (ATP) | d (NADH) | e (CO_2_) |
| AtoB + AdhEr route | butanol (C_4_H_10_O) | 0 | 1 | 2 | 0 | 2 |
| NphT7 + AdhEr route | butanol (C_4_H_10_O) | 0 | 1 | 1 | 0 | 3 |
| AtoB + TPC7 route | butanol (C_4_H_10_O) | 1 | 1 | 1 | 1 | 2 |
| NphT7 + TPC7 route | butanol (C_4_H_10_O) | 1 | 1 | 0 | 1 | 3 |
| FAS + TPC7 route | butanol (C_4_H_10_O) | 2 | 1 | 0 | 2 | 4 |
| AtoB route | crotonate (C_4_H_6_O_2_) | 0 | 1 | 2 | 3 | 2 |
| AtoB route | butyrate (C_4_H_8_O_2_) | 0 | 1 | 2 | 2 | 2 |
| AtoB route | butyraldehyde (C_4_H_8_O) | 0 | 1 | 2 | 1 | 2 |
