# Supplementary material for: In silico co-factor balance estimation using constraint-based modelling informs metabolic engineering in Escherichia coli
Source: PLoS Comput Biol. 2020 Aug 10;16(8):e1008125. doi: 10.1371/journal.pcbi.1008125 (PMC7440669; doi:10.1371/journal.pcbi.1008125)
Supplement: S12 Table — Stoichiometric coefficients from S9 Table were normalized per carbon-mol of glucose. (DOCX) [file pcbi.1008125.s012.docx]

| **Table S12 \| Normalized pathway coefficients a (NADPH), b (product), c (ATP), d (NADH) and e (CO2) of all butanol and butanol precursor pathways.** Stoichiometric coefficients from Table S9 were normalized per carbon-mol of glucose. | | | | | |
| --- | --- | --- | --- | --- | --- |
| Pathway | a (NADPH) | b (product) | c (ATP) | d (NADH) | e (CO_2_) |
| AtoB + AdhEr route | 0.000 | 0.167 | 0.333 | 0.000 | 0.333 |
| NphT7 + AdhEr route | 0.000 | 0.167 | 0.167 | 0.000 | 0.500 |
| AtoB + TPC7 route | 0.167 | 0.167 | 0.167 | 0.167 | 0.333 |
| NphT7 + TPC7 route | 0.167 | 0.167 | 0.000 | 0.167 | 0.500 |
| FAS + TPC7 route | 0.333 | 0.167 | 0.000 | 0.333 | 0.667 |
| AtoB route | 0.000 | 0.167 | 0.333 | 0.500 | 0.333 |
| AtoB route | 0.000 | 0.167 | 0.333 | 0.333 | 0.333 |
| AtoB route | 0.000 | 0.167 | 0.333 | 0.167 | 0.333 |
