# Supplementary material for: In silico co-factor balance estimation using constraint-based modelling informs metabolic engineering in Escherichia coli
Source: PLoS Comput Biol. 2020 Aug 10;16(8):e1008125. doi: 10.1371/journal.pcbi.1008125 (PMC7440669; doi:10.1371/journal.pcbi.1008125)
Supplement: S13 Table — (DOCX) [file pcbi.1008125.s013.docx]

| **Table S13 \| Upper and lower bound constraints derived from 13C- labelled data [42]** | | | |  |
| --- | --- | --- | --- | --- |
| **Reaction Name** | **Lower Bound** | **Upper Bound** | **Range** | |
| PFK | 2.843 | 25.962 | 23.119 | |
| GAPD | 2.721 | 15.845 | 13.124 | |
| PGK | 2.721 | 15.845 | 13.124 | |
| PYK | -0.273 | 3.326 | 3.599 | |
| G6PDH2r | 0.019 | 2.695 | 2.676 | |
| GND | 0 | 2.605 | 2.605 | |
| PDH | 1.846 | 11.2 | 9.354 | |
| ICDHyr | 0 | 29 | 29 | |
| AKGDH | 0 | 1.512 | 1.512 | |
| SUCOAS | -0.061 | 1.366 | 1.427 | |
| MDH | 0.356 | 3.445 | 3.089 | |
| ME2 | 0 | 0.044 | 0.044 | |
| ME1 | 0 | 0.302 | 0.302 | |
| PPCK | 0 | 1.058 | 1.058 | |
| ACKr | -0.076 | 7.69 | 7.766 | |
| ATPS4r | 7.417 | 25.821 | 18.404 | |
| THD2 | -0.984 | 8.058 | 9.042 | |
