# Supplementary material for: In silico co-factor balance estimation using constraint-based modelling informs metabolic engineering in Escherichia coli
Source: PLoS Comput Biol. 2020 Aug 10;16(8):e1008125. doi: 10.1371/journal.pcbi.1008125 (PMC7440669; doi:10.1371/journal.pcbi.1008125)
Supplement: S14 Table — (DOCX) [file pcbi.1008125.s014.docx]

| **Table S14 \| Upper and lower bound constraints derived from Flux Variability Analysis (fraction of optimum = 0.972) [19]** | | | |
| --- | --- | --- | --- |
| **Reaction Name** | **Lower Bound** | **Upper Bound** | **Range** |
| PFK | 4.981 | 11.906 | 6.925 |
| GAPD | 13.588 | 17.774 | 4.186 |
| PGK | 13.588 | 17.774 | 4.186 |
| PYK | 0 | 7.626 | 7.626 |
| G6PDH2r | 0 | 12.559 | 12.559 |
| GND | 0 | 12.559 | 12.559 |
| PDH | 6.575 | 15.254 | 8.679 |
| ICDHyr | 0.903 | 8.178 | 7.275 |
| AKGDH | 0 | 7.274 | 7.274 |
| SUCOAS | 0 | 7.274 | 7.274 |
| MDH | 2.431 | 10.7 | 8.269 |
| ME2 | 0 | 5.007 | 5.007 |
| ME1 | 0 | 3.641 | 3.641 |
| PPCK | 0 | 4.155 | 4.155 |
| ACKr | -1.093 | 0 | 1.093 |
| ATPS4r | 34.67 | 43.043 | 8.373 |
| THD2 | 0 | 12.559 | 12.559 |
