# Supplementary material for: In silico co-factor balance estimation using constraint-based modelling informs metabolic engineering in Escherichia coli
Source: PLoS Comput Biol. 2020 Aug 10;16(8):e1008125. doi: 10.1371/journal.pcbi.1008125 (PMC7440669; doi:10.1371/journal.pcbi.1008125)
Supplement: S15 Table — (DOCX) [file pcbi.1008125.s015.docx]

| **Table S15 \| Upper and lower bound constraints derived from MOMA [14]** | | | |
| --- | --- | --- | --- |
| **Reaction Name** | **Lower Bound** | **Upper Bound** | **Range** |
| PFK | 0 | 8.904 | 8.902 |
| GAPD | 0 | 17.257 | 17.257 |
| PGK | 0 | 17.257 | 17.257 |
| PYK | 0.329 | 4.453 | 4.124 |
| G6PDH2r | 0 | 21.332 | 21.332 |
| GND | 0 | 21.332 | 21.332 |
| PDH | 0 | 10.906 | 10.906 |
| ICDHyr | 0 | 7.82 | 7.82 |
| AKGDH | 0 | 6.932 | 6.932 |
| SUCOAS | 0 | 6.932 | 0 |
| MDH | 0 | 5.35 | 5.35 |
| ME2 | 0 | 1.582 | 1.582 |
| ME1 | 0 | 0.795 | 0.795 |
| PPCK | 0 | 50.85 | 50.85 |
| ACKr | 0 | 0 | 0 |
| ATPS4r |  | 5.573 | 43.269 |
| THD2 | 0 | 5.604 | 5.604 |
