# Supplementary material for: In silico co-factor balance estimation using constraint-based modelling informs metabolic engineering in Escherichia coli
Source: PLoS Comput Biol. 2020 Aug 10;16(8):e1008125. doi: 10.1371/journal.pcbi.1008125 (PMC7440669; doi:10.1371/journal.pcbi.1008125)
Supplement: S16 Table — Solutions were simulated under aerobic conditions and optimized for their selected objectives using pFBA: the wild type was optimized for biomass formation whilst the engineered models were optimized for target production. (DOCX) [file pcbi.1008125.s016.docx]

| **Table S16 \| pFBA flux distributions of wild type and engineered models constrained using 13-C MFA data.** Solutions were simulated under aerobic conditions and optimized for their selected objectives using pFBA: the wild type was optimized for biomass formation whilst the engineered models were optimized for target production. | | | | | | | | | | |
| --- | --- | --- | --- | --- | --- | --- | --- | --- | --- | --- |
|  | **WT** | **BuOH-0** | **BuOH-1** | **tpcBuOH** | **BuOH-2** | **fasBuOH** | **CROT** | **BUTYR** | **BUTAL** |  |
| ACKr | -0.076 |  |  |  |  |  |  |  |  |  |
| ACONT | 4.623 | 0.382 | 0.381 | 0.382 | 0.63 | 0.707 | 0.381 | 0.382 | 0.382 |  |
| ADK1 |  |  |  | 3.595 | 3.595 | 3.595 |  |  |  |  |
| AKGDH | 0.061 | 0.061 | 0.061 | 0.061 | 0.061 | 0.061 | 0.061 | 0.061 | 0.061 |  |
| AKGt2r | -2.132 |  |  |  | -0.248 | -0.326 |  |  |  |  |
| ATPM | 7.6 | 15.024 | 11.429 | 7.834 | 7.6 | 7.6 | 18.143 | 15.024 | 15.024 |  |
| ATPS4r | 25.821 | 7.417 | 7.417 | 7.417 | 10.529 | 10.452 | 10.536 | 7.417 | 7.417 |  |
| Biomass | 0.524 |  |  |  |  |  |  |  |  |  |
| CS | 4.623 | 0.382 | 0.381 | 0.382 | 0.63 | 0.707 | 0.381 | 0.382 | 0.382 |  |
| CYTBD | 29.476 | 7.192 | 7.192 | 5.754 | 8.493 | 8.803 | 12.238 | 8.643 | 7.911 |  |
| ENO | 14.113 | 7.571 | 7.571 | 7.571 | 8.068 | 8.223 | 7.571 | 7.571 | 7.571 |  |
| FBA | 7.19 | 3.352 | 3.351 | 3.352 | 3.6 | 3.677 | 3.352 | 3.352 | 3.352 |  |
| FBP |  |  |  |  |  |  |  |  |  |  |
| FUM | 1.926 | 0.382 | 0.381 | 0.382 | 0.381 | 0.382 | 0.381 | 0.382 | 0.382 |  |
| G6PDH2r | 2.605 | 2.605 | 2.605 | 2.605 | 2.605 | 2.605 | 2.605 | 2.605 | 2.605 |  |
| GAPD | 14.898 | 7.571 | 7.571 | 7.571 | 8.068 | 8.223 | 7.571 | 7.571 | 7.571 |  |
| GLCpts | 8.58 | 4.22 | 4.22 | 4.22 | 4.468 | 4.546 | 4.22 | 4.22 | 4.22 |  |
| GND | 2.605 | 2.605 | 2.605 | 2.605 | 2.605 | 2.605 | 2.605 | 2.605 | 2.605 |  |
| ICDHyr | 2.758 | 0.061 | 0.061 | 0.061 | 0.309 | 0.387 | 0.061 | 0.061 | 0.061 |  |
| ICL | 1.865 | 0.32 | 0.321 | 0.32 | 0.321 | 0.321 | 0.321 | 0.32 | 0.32 |  |
| MALS | 1.865 | 0.32 | 0.321 | 0.32 | 0.321 | 0.321 | 0.321 | 0.32 | 0.32 |  |
| MDH | 3.445 | 0.356 | 0.356 | 0.356 | 0.356 | 0.356 | 0.356 | 0.356 | 0.356 |  |
| ME1 | 0.302 | 0.302 | 0.302 | 0.302 | 0.302 | 0.302 | 0.302 | 0.302 | 0.302 |  |
| ME2 | 0.044 | 0.044 | 0.044 | 0.044 | 0.044 | 0.044 | 0.044 | 0.044 | 0.044 |  |
| NADH11 | 27.55 | 6.811 | 6.811 | 5.373 | 8.111 | 8.421 | 11.856 | 8.262 | 7.53 |  |
| NADTRHD |  | 4.331 | 4.331 | 0.736 | 0.985 |  | 5.315 | 4.363 | 4.331 |  |
| PDH | 8.53 | 7.585 | 7.585 | 6.147 | 8.14 | 8.218 | 1.846 | 1.846 | 4.709 |  |
| PFK | 7.19 | 3.352 | 3.351 | 3.352 | 3.6 | 3.677 | 3.352 | 3.352 | 3.352 |  |
| PFL |  | 0.307 | 0.307 | 1.745 |  |  | 6.046 | 6.046 | 3.183 |  |
| PGI | 5.867 | 1.615 | 1.615 | 1.615 | 1.863 | 1.941 | 1.615 | 1.615 | 1.615 |  |
| PGK | -14.898 | -7.571 | -7.571 | -7.571 | -8.068 | -8.223 | -7.571 | -7.571 | -7.571 |  |
| PGL | 2.605 | 2.605 | 2.605 | 2.605 | 2.605 | 2.605 | 2.605 | 2.605 | 2.605 |  |
| PGM | -14.113 | -7.571 | -7.571 | -7.571 | -8.068 | -8.223 | -7.571 | -7.571 | -7.571 |  |
| PPC | 2.115 | 0.026 | 0.025 | 0.026 | 0.274 | 0.351 | 0.025 | 0.026 | 0.026 |  |
| PYK | 3.146 | 3.326 | 3.326 | 3.326 | 3.326 | 3.326 | 3.326 | 3.326 | 3.326 |  |
| RPE | 1.36 | 1.737 | 1.737 | 1.737 | 1.737 | 1.737 | 1.737 | 1.737 | 1.737 |  |
| RPI | -1.245 | -0.868 | -0.868 | -0.868 | -0.868 | -0.868 | -0.868 | -0.868 | -0.868 |  |
| SUCD1i | 1.926 | 0.382 | 0.381 | 0.382 | 0.381 | 0.382 | 0.381 | 0.382 | 0.382 |  |
| SUCD4 | 1.926 | 0.382 | 0.381 | 0.382 | 0.381 | 0.382 | 0.381 | 0.382 | 0.382 |  |
| SUCOAS | -0.061 | -0.061 | -0.061 | -0.061 | -0.061 | -0.061 | -0.061 | -0.061 | -0.061 |  |
| TALA | 0.775 | 0.868 | 0.868 | 0.868 | 0.868 | 0.868 | 0.868 | 0.868 | 0.868 |  |
| THD2 | 1.545 | -0.984 | -0.984 | -0.984 | -0.984 | 1.549 |  | -0.952 | -0.984 |  |
| TKT1 | 0.775 | 0.868 | 0.868 | 0.868 | 0.868 | 0.868 | 0.868 | 0.868 | 0.868 |  |
| TKT2 | 0.585 | 0.868 | 0.868 | 0.868 | 0.868 | 0.868 | 0.868 | 0.868 | 0.868 |  |
| TPI | 7.19 | 3.352 | 3.351 | 3.352 | 3.6 | 3.677 | 3.351 | 3.352 | 3.352 |  |
| HCO3E |  |  | 3.595 |  | 3.595 | 3.595 |  |  |  |  |
| ACCOAC |  |  | 3.595 |  | 3.595 | 3.595 |  |  |  |  |
| NPHT7 |  |  | 3.595 |  | 3.595 |  |  |  |  |  |
| BUT1 |  | 3.595 |  | 3.595 |  |  | 3.595 | 3.595 | 3.595 |  |
| BUT2 |  | 3.595 | 3.595 | 3.595 | 3.595 |  | 3.595 | 3.595 | 3.595 |  |
| BUT3 |  | 3.595 | 3.595 | 3.595 | 3.595 |  | 3.595 | 3.595 | 3.595 |  |
| BUT4 |  | 3.595 | 3.595 | 3.595 | 3.595 |  |  | 3.595 | 3.595 |  |
| BTBTAC |  |  |  | 3.595 | 3.595 |  |  | 3.595 |  |  |
| CAR |  |  |  | 3.595 | 3.595 | 3.595 |  |  |  |  |
| BUT5 |  | 3.595 | 3.595 |  |  |  |  |  | 3.595 |  |
| BUT6 |  | 3.595 | 3.595 | 3.595 | 3.595 | 3.595 |  |  |  |  |
| BTOH_tr |  | 3.595 | 3.595 | 3.595 | 3.595 | 3.595 |  |  |  |  |
| BTOH_sink |  | 3.595 | 3.595 | 3.595 | 3.595 | 3.595 |  |  |  |  |
| MCOATA |  |  |  |  |  | 3.595 |  |  |  |  |
| KAS15 |  |  |  |  |  | 3.595 |  |  |  |  |
| 3OAR40 |  |  |  |  |  | 3.595 |  |  |  |  |
| 3HAD40 |  |  |  |  |  | 3.595 |  |  |  |  |
| EAR40x |  |  |  |  |  | 3.595 |  |  |  |  |
| 5_BUT1 |  |  |  |  |  | 3.595 |  |  |  |  |
| B2CTCRO |  |  |  |  |  |  | 3.595 |  |  |  |
| CROAC_tr |  |  |  |  |  |  | 3.595 |  |  |  |
| CROT_sink |  |  |  |  |  |  | 3.595 |  |  |  |
| BTAC_tr |  |  |  |  |  |  |  | 3.595 |  |  |
| BTAC_sink |  |  |  |  |  |  |  | 3.595 |  |  |
| BTAL_tr |  |  |  |  |  |  |  |  | 3.595 |  |
| BTAL_sink |  |  |  |  |  |  |  |  | 3.595 |  |
