# Supplementary material for: In silico co-factor balance estimation using constraint-based modelling informs metabolic engineering in Escherichia coli
Source: PLoS Comput Biol. 2020 Aug 10;16(8):e1008125. doi: 10.1371/journal.pcbi.1008125 (PMC7440669; doi:10.1371/journal.pcbi.1008125)
Supplement: S17 Table — Solutions were simulated under aerobic conditions and optimized for target production using pFBA. (DOCX) [file pcbi.1008125.s017.docx]

| **Table S17 \| pFBA flux distributions of engineered models constrained using 13-C MFA data and additional manual constraining of high-flux futile reactions.** Solutions were simulated under aerobic conditions and optimized for target production using pFBA. | | | | | | |
| --- | --- | --- | --- | --- | --- | --- |
|  | **BuOH-0** | **BuOH-1** | **tpcBuOH** | **CROT** | **BUTYR** | **BUTAL** |
| Reactions capped | ATPM, FBP | ATPM, FBP | ATPM, PPCK, FBP | ATPM, FBP | ATPM, FBP | ATPM, FBP |
| ACONT | 0.519 | 0.442 | 0.387 | 0.519 | 0.519 | 0.519 |
| ADK1 |  |  | 3.595 |  |  |  |
| AKGDH | 0.061 | 0.061 | 0.061 | 0.061 | 0.061 | 0.061 |
| ATPM | 7.6 | 7.6 | 7.6 | 7.6 | 7.6 | 7.6 |
| ATPS4r | 7.417 | 7.417 | 7.417 | 7.417 | 7.417 | 7.417 |
| Biomass | 0.128 | 0.056 | 0.005 | 0.128 | 0.128 | 0.128 |
| CS | 0.519 | 0.442 | 0.387 | 0.519 | 0.519 | 0.519 |
| CYTBD | 7.559 | 7.352 | 5.768 | 12.095 | 8.994 | 8.276 |
| ENO | 8.967 | 8.18 | 7.623 | 8.967 | 8.967 | 8.967 |
| FBA | 4.188 | 3.716 | 3.382 | 4.188 | 4.188 | 4.188 |
| FUM | 0.382 | 0.382 | 0.382 | 0.381 | 0.382 | 0.382 |
| G6PDH2r | 2.605 | 2.605 | 2.605 | 2.605 | 2.605 | 2.605 |
| GAPD | 9.158 | 8.263 | 7.63 | 9.158 | 9.158 | 9.158 |
| GLCpts | 5.183 | 4.64 | 4.255 | 5.183 | 5.183 | 5.183 |
| GND | 2.605 | 2.605 | 2.605 | 2.605 | 2.605 | 2.605 |
| ICDHyr | 0.199 | 0.121 | 0.066 | 0.199 | 0.199 | 0.199 |
| ICL | 0.32 | 0.32 | 0.32 | 0.321 | 0.32 | 0.32 |
| MALS | 0.32 | 0.32 | 0.32 | 0.321 | 0.32 | 0.32 |
| MDH | 0.356 | 0.356 | 0.356 | 0.356 | 0.356 | 0.356 |
| ME1 | 0.302 | 0.302 | 0.302 | 0.302 | 0.302 | 0.302 |
| ME2 | 0.044 | 0.044 | 0.044 | 0.044 | 0.044 | 0.044 |
| NADH11 | 7.177 | 6.971 | 5.387 | 11.713 | 8.612 | 7.895 |
| NADTRHD | 2.14 | 3.376 | 0.656 | 8.1 | 2.14 | 2.14 |
| PDH | 8.072 | 7.797 | 6.165 | 1.846 | 2.332 | 5.202 |
| PFK | 4.188 | 3.716 | 3.382 | 4.188 | 4.188 | 4.188 |
| PFL | 0.421 | 0.357 | 1.749 | 6.647 | 6.161 | 3.291 |
| PGI | 2.552 | 2.023 | 1.649 | 2.552 | 2.552 | 2.552 |
| PGK | -9.158 | -8.263 | -7.63 | -9.158 | -9.158 | -9.158 |
| PGL | 2.605 | 2.605 | 2.605 | 2.605 | 2.605 | 2.605 |
| PGM | -8.967 | -8.18 | -7.623 | -8.967 | -8.967 | -8.967 |
| PPC | 1.45 | 1.243 | 0.039 | 1.45 | 1.45 | 1.45 |
| PPCK | 1.058 | 1.058 |  | 1.058 | 1.058 | 1.058 |
| PYK | 3.326 | 3.326 | 3.326 | 3.326 | 3.326 | 3.326 |
| RPE | 1.645 | 1.697 | 1.733 | 1.645 | 1.645 | 1.645 |
| RPI | -0.96 | -0.908 | -0.872 | -0.96 | -0.96 | -0.96 |
| SUCD1i | 0.382 | 0.382 | 0.382 | 0.381 | 0.382 | 0.382 |
| SUCD4 | 0.382 | 0.382 | 0.382 | 0.381 | 0.382 | 0.382 |
| SUCOAS | -0.061 | -0.061 | -0.061 | -0.061 | -0.061 | -0.061 |
| TALA | 0.845 | 0.858 | 0.867 | 0.845 | 0.845 | 0.845 |
| THD2 | -0.984 | -0.984 | -0.984 | 4.975 | -0.984 | -0.984 |
| TKT1 | 0.845 | 0.858 | 0.867 | 0.845 | 0.845 | 0.845 |
| TKT2 | 0.799 | 0.838 | 0.866 | 0.799 | 0.799 | 0.799 |
| TPI | 4.188 | 3.716 | 3.382 | 4.188 | 4.188 | 4.188 |
| HCO3E |  | 3.592 |  |  |  |  |
| ACCOAC |  | 3.592 |  |  |  |  |
| NPHT7 |  | 3.592 |  |  |  |  |
| BUT1 | 3.587 |  | 3.595 | 3.587 | 3.587 | 3.587 |
| BUT2 | 3.587 | 3.592 | 3.595 | 3.587 | 3.587 | 3.587 |
| BUT3 | 3.587 | 3.592 | 3.595 | 3.587 | 3.587 | 3.587 |
| BUT4 | 3.587 | 3.592 | 3.595 |  | 3.587 | 3.587 |
| BTBTAC |  |  | 3.595 |  | 3.587 | 3.587 |
| CAR |  |  | 3.595 |  |  |  |
| BUT5 | 3.587 | 3.592 |  |  |  |  |
| BUT6 | 3.587 | 3.592 | 3.595 |  |  |  |
| BTOH_tr | 3.587 | 3.592 | 3.595 |  |  |  |
| BTOH_sink | 3.587 | 3.592 | 3.595 |  |  |  |
| B2CTCRO |  |  |  | 3.587 |  |  |
| CROAC_tr |  |  |  | 3.587 |  |  |
| CROT_sink |  |  |  | 3.587 |  |  |
| BTAC_tr |  |  |  |  | 3.587 |  |
| BTAC_sink |  |  |  |  | 3.587 |  |
| BTAL_tr |  |  |  |  |  | 3.587 |
| BTAL_sink |  |  |  |  |  | 3.587 |
