# Supplementary material for: In silico co-factor balance estimation using constraint-based modelling informs metabolic engineering in Escherichia coli
Source: PLoS Comput Biol. 2020 Aug 10;16(8):e1008125. doi: 10.1371/journal.pcbi.1008125 (PMC7440669; doi:10.1371/journal.pcbi.1008125)
Supplement: S18 Table — (DOCX) [file pcbi.1008125.s018.docx]

| **Table S18 \| Reactions added to the Core Model of *E.coli* to implement biosynthetic production of butanol and butanol precursors.** | | | | | |
| --- | --- | --- | --- | --- | --- |
| **Reaction ID** | **Reaction name** | **Gene(s)** | **EC** | **Reaction** | **Model(s)** |
| ACCOAC | Acetoacetyl-CoA carboxylase | *acc* | 6.4.1.2 | accoa_c + atp_c + hco3_c => adp_c + malcoa_c + pi_c | BuOH-1, BuOH-2, fasBuOH |
| ACPS1 | Acyl-ACP synthetase | *aas* | 6.2.1.20 | apoACP_c + coa_c --> ACP_c + h_c + pap_c | fasBuOH |
| CAR | Carboxylic acid reductase | *car* | 1.2.99.6 | atp_c + btac_c + h_c + nadph_c --> amp_c + btal_c + h2o_c + nadp_c + 2.0 pi_c | tpcBuOH, BuOH-2, fasBuOH |
| BPNT | 3’(2’),5-biphosphate nucleotidase | *cysq* | 3.1.3.7 | h2o_c + pap_c --> amp_c + pi_c | fasBuOH |
| BTBTAC | Acyl-CoA thioester hydrolase | *ycia* | 3.1.2.20 | butcoa_c + h2o_c --> btac_c + coa_c | tpcBuOH, buOH-2, BUTYR |
| BUT1 | Acetyl-CoA acetyltransferase | *atob* | 2.3.1.9 | 2.0 accoa_c => acoa_c + coa_c | BuOH-0, tpcBuOH, fasBuOH, CROT, BUTYR, BUTAL |
| BUT2 | 3-hydroxybutyryl-CoA dehydrogenase | *hbd* | 1.1.1.157 | acoa_c + h_c + nadh_c --> 3hbcoa_c + nad_c | BuOH-0, BuOH-1, BuOH-2,  tpcBuOH, CROT, BUTYR, BUTAL |
| BUT3 | 3-hydroxybutyryl-CoA dehydratase | *crt* | 4.2.1.55 | 3hbcoa_c --> b2coa_c + h2o_c | BuOH-0, BuOH-1,  tpcBuOH, BuOH-2, CROT, BUTYR, BUTAL |
| BUT4 | Trans-2-enoyl-CoA reductase | *ter* | 1.3.1.44 | b2coa_c + h_c + nadh_c --> butcoa_c + nad_c | BuOH-0, BuOH-1, tpcBuOH, BuOH-2, BUTYR, BUTAL |
| BUT5 | Aldehyde-alcohol dehydrogenase | *adhe2* | 1.1.1.1 | butcoa_c + h_c + nadh_c --> btal_c + coa_c + nad_c | BuOH-0, BuOH-1, BUTAL |
| BUT6 | Aldehyde reductase | *ahr* | 1.1.1.2 | btal_c + h_c + nadh_c --> nad_c + nbutanol_c | BuOH-0, BuOH-1, tpcBuOH, BuOH-2, fasBuOH |
| B2CTCRO | Acyl-CoA thioester hydrolase | N. A | 3.1.2.20 | b2coa_c + h2o_c --> coa_c + croac_c | CROT |
| EAR40x | Enoyl-ACP reductase |  | 1.3.1.9 | M_but2eACP_c + h_c + nadh_c --> butACP_c + nad_c | fasBuOH |
| HCO3E | Carbonic anhydrase | *cynt* | 4.2.1.1 | co2_c + h2o_c --> h_c + hco3_c | BuOH-1, BuOH-2, fasBuOH |
| KAS15 | Beta-ketoacyl-ACP synthase II | *fabf* | 2.3.1.41 | accoa_c + h_c + malACP_c --> actACP_c + co2_c + coa_c | fasBuOH |
| MCOATA | Malonyl-CoA-ACP transacylase | *fabD* | 2.3.1.39 | ACP_c + malcoa_c <=> coa_c + malACP_c | fasBuOH |
| NPHT7 | Acetoacetyl-CoA synthase | *npht7* | 2.3.1.194 | accoa_c + malcoa_c --> aacoa_c + co2_c + coa_c | BuOH-1, BuOH-2, |
| 3HAD40 | 3-hydroxyacyl-ACP dehydratase | *fabF* | 4.2.1.59 | 3haACP_c --> M_but2eACP_c + h2o_c | fasBuOH |
| 3OAR40 | 3-oxoacyl-ACP reductase | *fabG* | 1.1.1.100 | actACP_c + h_c + nadph_c <=> 3haACP_c + nadp_c | fasBuOH |
| 5_BUT1 | Acyl-ACP thioesterase | *aat* |  | butACP_c + h2o_c --> ACP_c + btac_c | fasBuOH |
| BTAC_tr | Butyric acid transport extracellular space |  |  | btac => e_btac | BUTYR |
| BTAC_sink | Butyric acid drain from model |  |  | e_btac => | BUTYR |
| BTAL_tr | Butyraldehyde transport extracellular space |  |  | btal => e_btal | BUTAL |
| BTAL_sink | Butyraldehyde drain from model |  |  | e_btal => | BUTAL |
| BTOH_tr | Butanol transport extracellular space |  |  | nbutanol_c --> n-butanol_e | BuOH-0, BuOH-1, tpcBuOH, BuOH-2, fasBuOH |
| BTOH_sink | Butanol drain from model |  |  | n-butanol_e --> | BuOH-0, BuOH-1, tpcBuOH,  BuOH-2, fasBuOH |
| CROAC_tr | Crotonate transport to extracellular space |  |  | croac => e_croac | CROT |
| CROAC_sink | Crotonate drain from model |  |  | e_croac => | CROT |
