# Supplementary material for: In silico co-factor balance estimation using constraint-based modelling informs metabolic engineering in Escherichia coli
Source: PLoS Comput Biol. 2020 Aug 10;16(8):e1008125. doi: 10.1371/journal.pcbi.1008125 (PMC7440669; doi:10.1371/journal.pcbi.1008125)
Supplement: S1 Fig — (DOCX) [file pcbi.1008125.s019.docx]

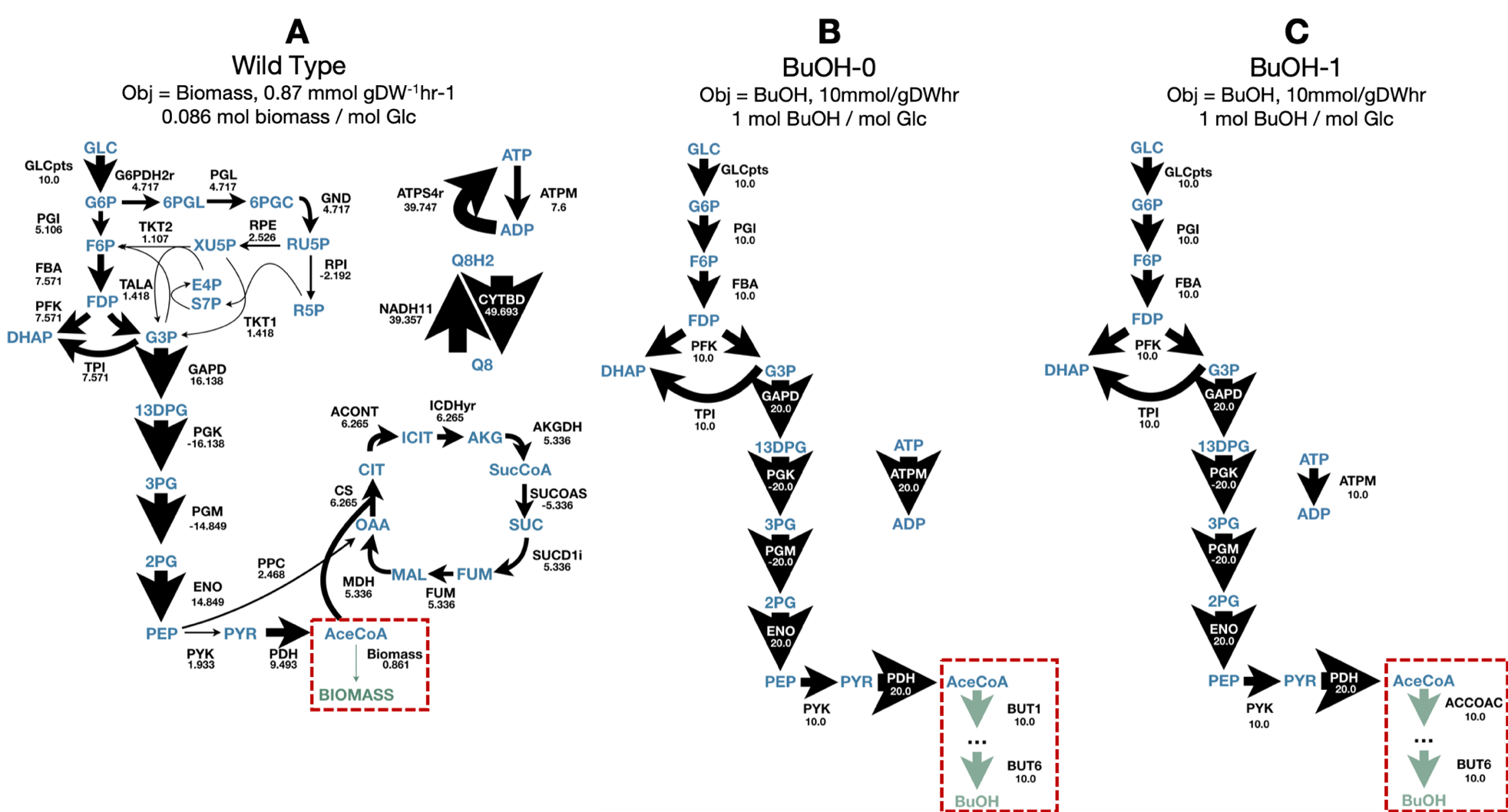


Figure S1 ***| pFBA flux distribution maps of unconstrained models under aerobic conditions***

**
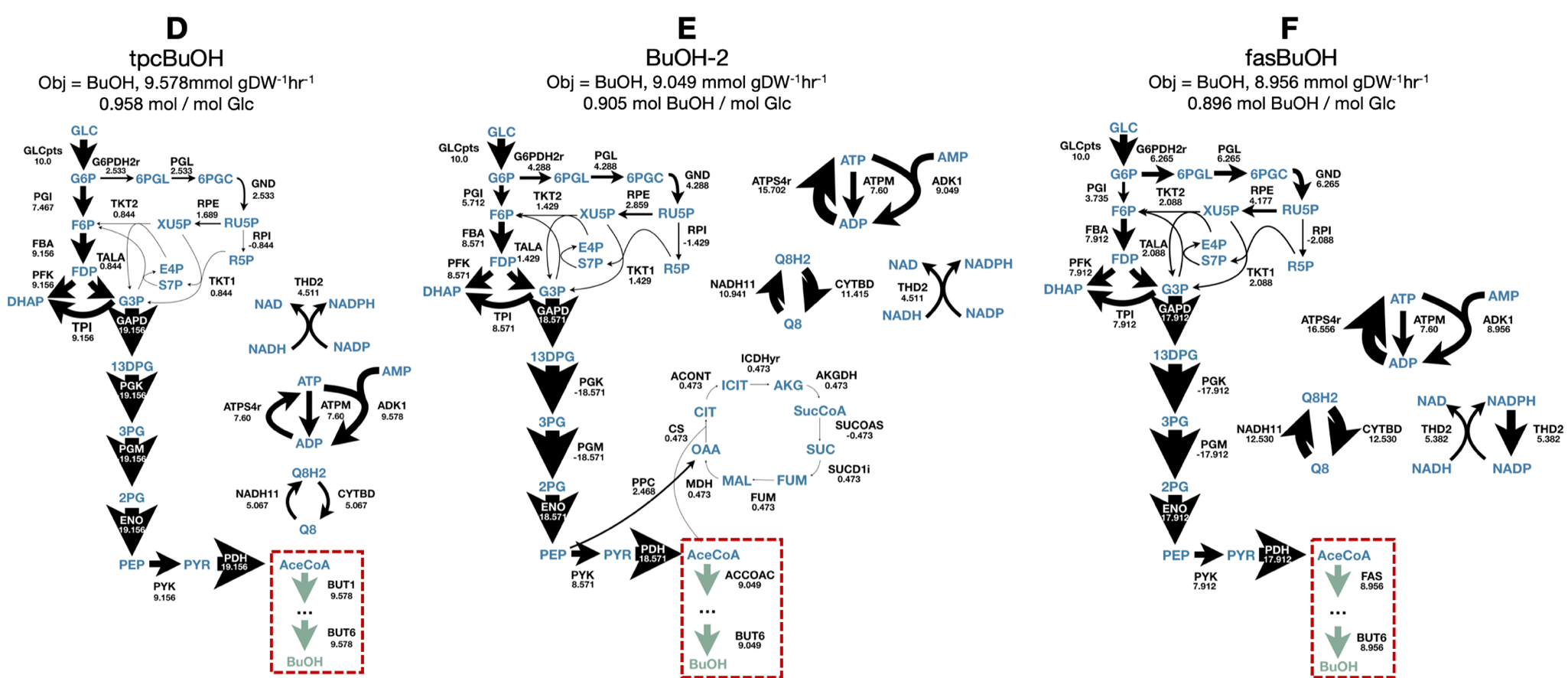
**

Figure S1 (continued) ***| pFBA flux distribution maps of unconstrained models under aerobic conditions***

***
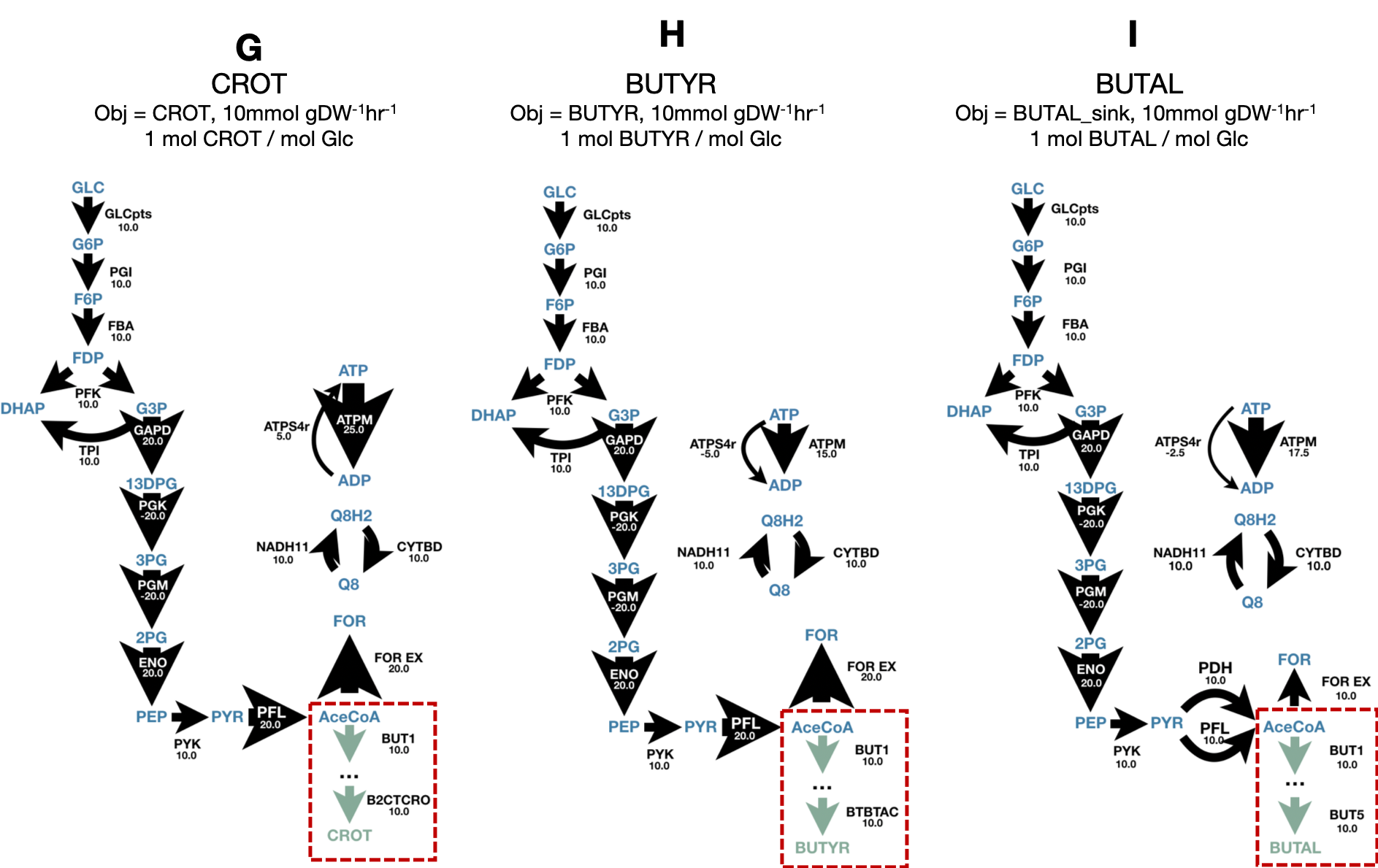
***

Figure S1 (continued) ***| pFBA flux distribution maps of unconstrained models under aerobic conditions***
