# Supplementary material for: In silico co-factor balance estimation using constraint-based modelling informs metabolic engineering in Escherichia coli
Source: PLoS Comput Biol. 2020 Aug 10;16(8):e1008125. doi: 10.1371/journal.pcbi.1008125 (PMC7440669; doi:10.1371/journal.pcbi.1008125)
Supplement: S2 Fig — Each model represents a unique pathway variant for butanol production, which has been manually curated and optimized for the selected objective under aerobic conditions. (A) BuOH-0, comprised of route AtoB + AdhE2; (B) BuOH-1, including reactions NphT7 + AdhE2; (C) tpcBuOH, made up of AtoB + TPC7; (D) fasBuOH, comprising reactions NphT7 + TPC7. (DOCX) [file pcbi.1008125.s020.docx]

*
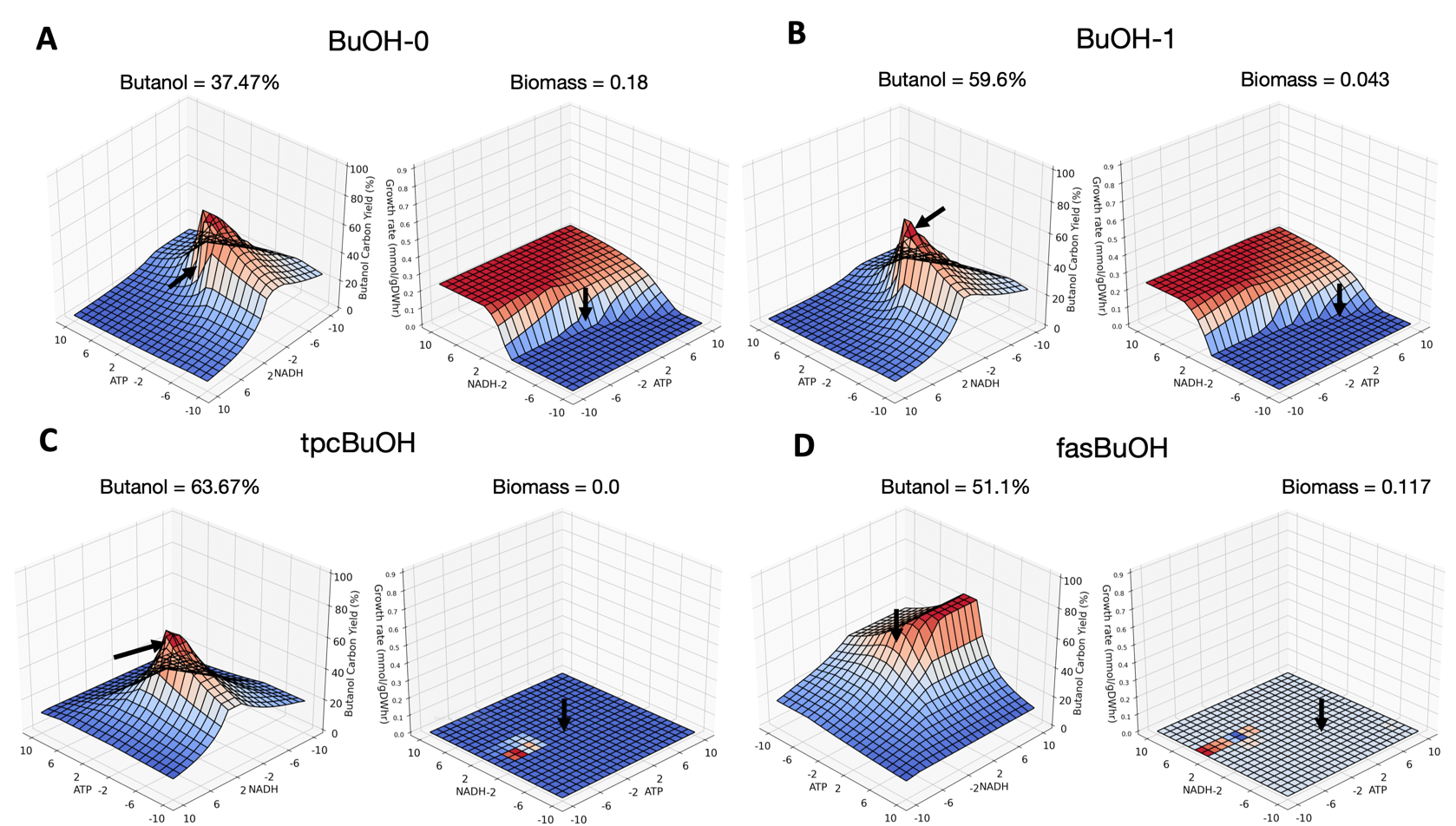
*

Figure S2 | ***butanol carbon yield (%) and biomass production rates (mmol gDW^-1^hr^-1^) of engineered E.coli strains in response to changes in ATP and NADH demands under anaerobic conditions****. Each model represents a unique pathway variant for butanol production, which has been manually curated and optimized for the selected objective under aerobic conditions. (A) BuOH-0, comprised of route AtoB + AdhE2; (B) BuOH-1, including reactions NphT7 + AdhE2; (C) tpcBuOH, made up of AtoB + TPC7; (D) fasBuOH, comprising reactions NphT7 + TPC7.*
