# Supplementary material for: In silico co-factor balance estimation using constraint-based modelling informs metabolic engineering in Escherichia coli
Source: PLoS Comput Biol. 2020 Aug 10;16(8):e1008125. doi: 10.1371/journal.pcbi.1008125 (PMC7440669; doi:10.1371/journal.pcbi.1008125)
Supplement: Attachment — Submitted filename: PLOS rebuttal letter.docx [file pcbi.1008125.s036.docx]

Department of Life Sciences

Imperial College London

SAF Building, room 503

SW7 2AZ, London, UK

Tel: +44 (0)20 7594 5213

[p.jones@imperial.ac.uk](mailto:p.jones@imperial.ac.uk)

[www.imperial.ac.uk/microbial-metabolic-engineering](http://www.imperial.ac.uk/microbial-metabolic-engineering)

**Patrik R. Jones** Ph.D.

Reader in Metabolic Engineering

Admissions Tutor Biochemistry

Dear Editor,

Thank you very much for reviewing our manuscript and for your patience during our revision process. We greatly appreciate your time and feedback. Most of the raised points were highly valid and we believe that in answering these questions and adapting the draft we have substantially improved the manuscript.

After receiving and carefully considering all your comments, we have revised the body of the manuscript and clarified the wording and ambiguities, carried out additional analyses, and introduced new supporting data that we believe answers the reviewers concerns and clarifies our work further.

In addition to the revisions made in response to reviewers’ comments, additional significant updates to our resubmission include:

- After careful consideration, we have amended the title of the manuscript to read “***In silico* co-factor balance estimation using constraint-based modelling informs metabolic engineering in *Escherichia coli***”. Given that we use FBA, pFBA, FVA, MOMA and CBA, we believe this title is more representative of the work performed.
- All our scripts, including the CBA protocol, have been updated to run on the latest versions of all packages, namely to be compatible with Python 3 and the latest version of COBRApy. We believe this was necessary to cater to the fast-developing nature of the Python community, in order to guarantee that the scripts can be easily implemented and run in the latest environments with minimal compatibility hurdles.
- We have submitted copies of all constraint-based scripts and the CBA algorithm, as requested.
- We are also providing copies (in SBML format) of all of the stoichiometric models generated for butanol and butanol precursor production in *E.coli.*

These changes have also been duly reflected in the manuscript.

We appreciate that the manuscript will now be re-evaluated in response to our re-submission. We look forward to receiving your response.

Yours sincerely,

Patrik R. Jones and Laura de Arroyo Garcia

**Rebuttal**

Responses are shown in blue and any changes made to the manuscript are highlighted in green (and also shown in the manuscript document as tracked changes)

**Reviewer #1**: In this paper, de Arroyo Garcia and Jones explore how cell metabolism can limit the yield of synthetic metabolic pathways. In the growing field of metabolic engineering, were cells are treated as potential “factories” to synthesize industrial compounds, it is important to consider the capability of the cells native metabolism to “mesh” with the demands of synthetic pathways, as these pathways will alter the homeostasis of cellular energy charge and redox.

The methodology is centred on the use of flux balance analysis, but with extensive manual curation of the reaction constraints. Throughout, various pathways to n-butanol, a non-native product in E. coli, are considered based on how their NADPH and ATP demands are met by the cell, and what the true pathway yields would be. The main technical novelty is a new script for tracking and categorizing how cellular ATP and NADPH are affected (produced or consumed) in the presence of the new pathway.

The concept is not altogether novel, as pointed out by the authors they are following concepts laid out by Dugar and Stephanopoulos 2011. However, this approach is useful in that it uses a core metabolic model instead of a pathway in isolation. The authors are thorough as well, they systematically “hunt” and constrain futile cycles as well as key reactions that appear to be bounded when analyzing experimental MFA data of knockout strains. This latter addition is interesting, as the MFA data suggest that cellular metabolism is not as flexible in adapting to knockouts as it appears from stoichiometric metabolic models. As a result, the limitations of FBA are systematically revealed and addressed in order to arrive at a more accurate prediction of a pathway’s actual yield.

Overall the paper is well conceived and well-written. It is somewhat specific to butanol, but there is a variety of butanol pathways considered that allow somewhat general conclusions to be drawn about expected pathway yields based on ATP and NADPH usages. The technical novelty is a bit lacking.

Specific points that could be addressed to improve the manuscript:

The authors use FBA with product maximization, and then record rates through various ATP and NADPH reactions. FBA gives one particular flux distribution out of many, would it have been better to use FVA here instead?

**Response 1:** This is an important point, and one which was raised also by the other reviewers. FBA was used in order to obtain data for the CBA under optimal conditions. An FVA was also carried out with all the models (Table S1) but no explanation was provided in submission 1. Even though theoretically many solutions may exist, as it turns out, regardless of what the objective function was, we found no or very little variation amongst the individual reactions, with most of the models. Two of the models did display variation in ~15% of the reactions (14/99 or 18/101) but none of these reactions were directly in the pathway towards butanol, and all were directly involved in futile co-factor cycling. The limited solution space in these models is most likely due to the fact that we used (i) a minimal core model, and because we (ii) focus on the optimal solution. Hence, for the purpose of this work, the single solution provided by FBA is highly likely to be sufficiently representative and is simpler to use. Once constraints from MFA or manual elimination of futile cycling is applied, there is only ever a single solution. To clarify this, we have added a paragraph to the section where the models are first characterized, section 2.1, and hasve also included in the supplement the FVA results of all models after manual curation, showing they all have a single solution (Tables S9 and S10).

*"The solution space of all models was investigated by flux variability analysis (FVA) [19] (Table S1, Supplement1). Considering only reactions accounted for by CBA, only a single solution was found with the wild type and models tpcBuOH, BuOH-2 and fasBuOH. In contrast, the butanol producing models BuOH-0 and BuOH-1 had varying flux ranges in 14 out of 99 and 18 out of 101 reactions, respectively. Notably, none of the reactions displaying multiple solutions were directly in the path towards butanol, and all were involved in futile co-factor cycles (see 2.4). This is interesting given that the models display no flux variability in their target product pathways, whilst the reactions that did show flux variability were all co-factor related reactions."*

Authors use product as cellular objective and then tabulate fluxes of ATP and NADPH-related reactions. However, there may be more realistic ways to estimate how the cell would respond to new pathways. The MOMA technique (Segre et al., 2002) is another way to estimate fluxes after network perturbation, such as addition of a biosynthetic pathway, while biomass remains the cellular objective. The authors could consider such alternative methods for comparison, to see if similar ATP and NADPH-consuming rates arise. Perhaps MOMA where a certain flux into production pathway is used as a constraint.

**Response 2:** We thank the reviewer for this idea. As the objective of our study initially was to implement a more flexible approach to Dugar-like analysis, FBA was selected as it provides information about the theoretically maximum pathway potential. As our study progressed, however, the observation that futile and obviously artificial co-factor cycles had substantial impact on the outcome raised the question of how to obtain more realistic data. We chose the path of exploring different methods to apply constraints, but indeed, MOMA is another attractive option. We felt it was important to respond to this idea, and also illustrate alternative approaches, and therefore incorporated a MOMA-based evaluation of the data generated by Long et al. (2019). The results are summarized in Figure 6A (section 2.6) where we compare the variability in measured vs. predicted flux ranges, across 15 different knock-outs, using both FVA and MOMA. In general, most of the reactions were still more plastic in computational predictions than what was measured experimentally. The discussion referring to this figure was extended in order to make it more clear, and to add a rationale for why MOMA was evaluated.

In Abstract and Introduction, authors refer to “flexibility” of computational predictions. Later in the manuscript it is clear they mean possible flux patterns to reach the same objective (FVA). I recommend defining what is meant by flexibility (perhaps plasticity?) as early as possible (preferably in Abstract).

**Response 3:** Very good point. Other reviewers also raised this point and mentioned this “flexibility” occurs as a result of the underdeterminacy of the FBA models, given that we are running them on no or very little constraints prior to manual curation or application of MFA constraints. In response to this, we have reworded the manuscript throughout to refer to this issue as a consequence of the underdetermined nature of the models, including the abstract. As follows: "*Predicted solutions were compromised by the excessively undetermined nature of the systems, displaying up to 11-fold greater flexibility in the range of reaction fluxes than experimentally measured by 13C-metabolic flux analysis (MFA)."*

The authors use various butanol pathways as case studies, and also attempt to generalize findings about the predicted yields of pathways with certain ATP and NADPH demands. The reliance on MFA data from multiple mutants could limit the approach to be only available for E. coli or yeast as host strains, but these are likely still useful to get an idea of how a synthetic pathway could be limited in different hosts.

**Response 4:** Indeed. Our hope is that other researchers can carry out CBA analyses with or without MFA data as constraints. In the case that MFA data is not available, manual constraints or MOMA could be considered instead. The aim of the present study was also to highlight some of the issues with FBA in the absence of constraints, in particular the appearance of futile cycles which cannot be overcome with alternative tools such as loopless FBA.

From the review file and SI, it was not clear if all scripts and models have been made publicly available. Missing are the different models and the Python scripts used to perform CBA. I encourage the authors to provide those.

**Response 5:** copies of the butanol-producing models (in SBML format) and of the CBA algorithm and supplementary code have now been duly submitted.

Looking at table 1, NADPH and NADH are lumped together when it comes to pathway requirements. How is this translated in the model? Is there one reaction for each cofactor? Please clarify.

**Response 6:** NADPH and NADH are assumed to be interchangeable for modelling purposes. This means that if a (hypothetical) biosynthetic pathway produces 1 NADPH and 1 NADH, the total redox produced will be +2 NAD(P)H, instead of each co-factor being considered separately.

Fig. 1 Could be helpful to have enzyme abbreviation directly on the figure

**Response 7:** Thank you for pointing this out. We have added the enzyme names to the figure.

Fig. 2 A) What does the thickness of the arrows mean? Is it proportional to the flux amplitude? If yes, please state under which condition CBA was performed, with WT or one of the “engineered” models?
B) Same as A), few flux values a represented but it is not clear where they come from

**Response 8:** Figure 2 is a toy example, a theoretical graphical representation of how CBA functions. The legend has now been revised to make this clear.

Fig. 2 and Fig.3 NADPH waste: In fig.2 caption, it says “waste category may also include reactions..” What are these other reactions?

**Response 9:** Thanks for identifying this unclarity. The legend in Fig. 2 has been modified as follows: “*The NAD(P)H waste category also accounts for reactions GND, PDH, AKGDH, ICDHyr, which* ***produce*** *NAD(P)H but simultaneously release CO_2_, and reactions such as LDH_D and AdhEr that* ***consume*** *NAD(P)H and release fermentation products”*

l.248 “Under aerobic conditions, solutions for the engineered models showed no biomass accumulation” Was it also the case in aerobic conditions?

**Response 10:** Yes that is correct, (see Fig 3C how there is no ATP or redox invested in the generation of biomass). We have modified l.255 to state *“Both under aerobic and anaerobic conditions, solutions for the engineered models showed no biomass accumulation”*

l.257 (and Fig. 3) If PDH is the major contributor of NADPH waste for ButOH-0, ButOH-1 and tpcBuOH, what is the major contributor of NADPH consumed in the NADPH waste?

**Response 11:** PDH produces NAD(P)H (by reduction of NAD+) but also liberates CO2, hence it is labelled as "waste". The explanation for the labelling in CBA is summarized in the toy illustration, Figure 2, where PDH is one of the examples used. In Table S2, all the fluxes are summarized, here we can see that PDH flux is 20 or almost 20 in all these models, i.e. ButOH-0, ButOH-1 and tpcBuOH.

The main consumption of the NAD(P)H produced by the PDH reaction (and that produced by GAPD which is labelled "produced" as there is no excretion product) are the four NAD(P)H-oxidizing steps in the butanol pathway. To clarify this, we revised the paragraph which previously was at line 257, as follows:

"*All butanol models relied on the glyceraldehyde-3-phosphate dehydrogenase (GAPDH and pyruvate dehydrogenase reaction (PDH) for the supply of redox. The PDH reaction is known to provide the extra redox needed for butanol production [20]. As illustrated in Fig. 3, PDH is labelled as "waste", because it contributes to the loss of carbon through CO_2_ release (hence the positive value observed for NAD(P)H waste under aerobic conditions, Figure 4A, yellow). However, it simultaneously supplies the target pathway a key limiting factor, NADH, which is essential to optimize flux towards butanol production."*

Also related to this point, maybe the authors can expand on why the NADPH waste is 0 in BUTYR (Fig.3 B/C) versus non-zero in BUTAL. After all, these two models are one reaction consuming ATP apart

**Response 12:** BUTYR and BUTAL are one NADH-consuming reaction apart, and their difference in NAD(P)H waste stems from the fact that additional NAD(P)H is required and PDH is the supplier. This is most easily seen by looking at Supplementary Table S2 and Supplementary Figure S1(Supplement), where it is noted that BUTAL generates the extra NADH required for butyraldehyde production by activating PDH (which is inactive in BUTYR, with flux instead directed through the PFL reaction which is redox-neutral). As explained under **Response 11**, PDH is classified as a waste reaction due to the carbon lost as CO_2_.

l.377 “After cycle detection,..” What is the cutoff value here to decide that this particular set of reactions is now actually a futile cycle?

**Response 13:** We identified futile cycles manually and systematically, by identifying new cyclic fluxes between reaction pairs (or more than 2 reactions in rare instances) that in all cases involved co-factor metabolism and appeared as a result of changes to the original model. Typically, reaction pairs presented identical (or proportional) fluxes, meaning in one direction the cofactor excess was burned (ATP) or chanelled electrons further (NAD(P)H). We only considered futile cycles with significant flux. In most, if not all, cases the cyclic flux was obvious, typically having a cyclic flux exceeding 5 mmol gDW-1hr-1.

The only situation in which we take into account a “cutoff” value per se was for the ATPM reaction, where a minimum of 7.6 mmol gDW^-1^ hr^-1^ is used for general ATP maintenance.

l.403 “BuOH-1 appears to be the most balanced..” most balanced in term of what metric? Redox balance? It would seem that the model having no flux towards biomass instead is actually the most balance redox-wise.

**Response 14:** Correct, this is also the point that Dugar & Stephanopoulos make and we try to emphasize in this paper. The sentence reviewer 1 highlights, however, was unclear and ambiguous. The relevant paragraph (section 2.4) was reworded in order to make it more clear:

*“In theory, an engineered organism having no ATP or redox flux towards biomass would be considered the most balanced, presumably because any ATP and redox are instead being invested in the maintenance of the biosynthetic pathway in question (or balanced through the release of fermentation products, as our results illustrate above). In reality, however, a viable engineered system will always produce some biomass. However, ideally, the system should produce just enough biomass to guarantee its own survival, but not so much that this is achieved at the detriment of target production. In this respect, from our analysis, we can conclude that BuOH-1 appears to be the most balanced pathway after manual constraints because, not only does it have the highest butanol efficiency (96.64%), but the CBA protocol indicates that this model also presents the lowest biomass yield, i.e. it is the engineered model which diverts the least amount of redox AND ATP towards biomass formation, and achieves the highest fluxes through the target production route.”*

Related to the above point, have the authors tried to include an artificial NADPH and/or an ATP sink reaction in the model to see how imbalanced the redox state is? It would be interesting to see if these reactions would have a non-zero flux in the model making no biomass for instance. It could also be used as a metric to determine redox imbalance in each model. l.427 Same as above artificial sink reaction could prove that. If you add an ATP sink reaction, how does the CBA analysis of NADPH vary and vice versa

**Response 15:** Agreed. Please refer to Figure 7 and section 2.7 where this idea has been implemented. The introduction in this section has now been reworded to better clarify the aims.

l.501 and paragraph. I think this is an unfair comparison between 13C-MFA and FVA variability. First, knocking-out a single gene would not probably push the reactions of the network to their maximal range as this is the case in silico. Secondly, this is certainly not the case with FBA as it predicts a long-term flux distribution (after many generations). Whereas the 13C-MFA data obtained from Havekorn et al. will provide flux data that should instead be compared to MOMA (Segre et al.).

**Response 16:** We agree and thank the reviewer for this idea, which we implemented in Figure 6 and heavily revised section 2.6. MOMA provides a flux distribution corresponding to a knockout strain, and approximates this to a reference model. It does not produce a range of fluxes however. To create a flux range for comparison with the ranges derived from MFA data, we needed to simulate the knockout strains in the flux dataset. The issue with this is that the Haverkorn dataset implemented knockouts of transcriptional factors, and then measured the effect of these on central metabolism with MFA. Because these knockouts could not be directly implemented in MOMA, we switched to the MFA dataset provided by the recent publication by Long et al. (2019) (doi: [10.1016/j.ymben.2019.08.003](https://doi.org/10.1016/j.ymben.2019.08.003)) a study that evaluates the effects of 20 knockouts (15 of which were in the model used in our study) of reactions in central carbon metabolism. Applying each of these knockouts as a separate MOMA simulation, we derived a flux range in each reaction across all 15 knockouts. Our results for this section have thus been considerably updated to fit and analyse this new data, which is shown in Figure 6. For comparison, the FVA data is still included, as it helps to provide context given that FBA is used in the CBA calculation. (FVA was calculated at 97% of the optimum, this is the deviation in growth rate found in the Long et al. study)

Fig.7 Great results to guide pathway engineering (on a pure stochiometric aspect). From this figure, it seems it would be possible to determine an “optimal” ATP/NADPH ratio for each product. I would also suggest to add a marker showing where each model stand in each sub-plot

**Response 17:** Thank you. The biomass yield and marker was added to Figure 7, as well as a final concluding paragraph to close this section.

l.607 Can the authors hypothesize on why no solutions exist for ATP produced >2 in Fig. 7 G & H. Since product is set as objective function, does this mean that no solution exist? It would be good to indicate that there is no flux solution (leave the area blank) rather than implying no flux to product.

**Response 18:** Because we included the biomass yield in the new version of Figure 7, anaerobic results have been moved to the Supplement, as Figure S2. Solutions do exist for ATP >2 for tpcBuOH and fasBuOH, however they are so small that they approximate zero in the figure.

**Reviewer #2**: The authors had a very interesting idea to explore cofactor balance and its relation to maximizing the production of compounds. The authors leveraged FBA, CBA, FBA, and MFA to interrogate the Ecoli core and 8 additional engineered models with different combinations of pathways that leads to the biosynthesis of butanol. Each model had a different demand for ATP and NAD(P)H and the authors looked at how those change in redox demand effected flux distribution and yield under aerobic and anaerobic conditions. I think the idea is interesting, however there are some concerns associated with the analysis.

1) The comparison between WT and engineered strains is not a fair comparison due to the difference in objective functions. Would the results change if all of the engineered strains were constrained to the growth rate of the WT, and then maximizing the production of butanol?

**Response 19:** We agree that models having different objective functions cannot be directly compared, therefore we refrained from making direct mathematical comparisons, and the WT model flux distribution was described as a reference of the “average” WT behaviour. E.g. (section 2.6) *"Models of each single knockout from Long et al. were optimized using either FBA or MOMA [14] with the wild type E.coli solution as a reference"*.

In response to this comment, we ran a new simulation where the biomass reaction of the engineered strains were fixed to 100%, 80%, 60%, 40%, 20% and 0% of the WT biomass.

**Flux variation according to biomass production.** The biomass reaction of all engineered models was fixed at 100%, 80%, 60%, 40%, 20% and 0% of the growth observed in the wild type. Models were then optimized for target production.

As expected, butanol production, biomass and also the fluxes across all ATP/NAD(P)H related reactions change gradually with the gradual change in biomass formation. Not surprisingly, there is no butanol production when biomass is fixed to 100% WT. The results illustrate that there is an undeniable relationship between changes in biomass, co-factor metabolism, and ultimately target production. Although this method of analysis could be extended to alternative methods of visualization or inclusion of constraints, we feel that it will result in an article that is trying to do too much, and that it will distract the reader and lose focus. Therefore, we suggest not to include it, nor any further elaborations upon it.

2) FVA not conducted across all engineered strains. Single FBA/CBA flux distributions were used to draw conclusions about how redox demand effects the over flux distribution of the network. However, many other solutions exist that yield the same objective value, the singular solution that the author presented and analyzed will change based on the algorithm and solver that was used to solve the linear programming problem. Although the yields may be correct, the CBA may yield an incomplete picture. How much did the cofactor contributions change if FVA was performed?

**Response 20:** Reviewer 1 made a similar comment. Please refer to **Response 1** for a discussion on this point.

3) NADPH and NADH are analyzed interchangeably, however pathways such as pentose phosphate replenishes NADPH and can contribute to the cofactor analysis.

**Response 21:** By analysing them “interchangeably” we mean that we do not make a distinction between NADH and NADPH when CBA is applied, given that the transhydrogenase SthA is allowed to freely channel electrons between the two co-factor pools. Hence, the consumption and production of these two co-factors are both accounted for in an overall “redox” category, instead of having two separate groups. However, their usage within the model will still be very much dependent on which co-factor is used by which reaction, i.e. NADPH generated by the PPP will be captured by CBA. We explain this in the methods section: *"CBA solely tracks ATP and NAD(P)H metabolism, while all other co-factors are excluded from the analysis. For modelling purposes, it assumes that NADH and NADPH are interchangeable, even though in reality NADH and NADPH are not biologically equivalent."*

**------------------------------------------------------**

**Reviewer #3**: This paper proposes a procedure to analyse the impact of metabolic engineering on the balance of co-factors, and use it to evaluate alternative metabolic engineering designs.

The results are interesting and the paper is well written.

However, the several computational procedures could be presented with more mathematical details:

1) In Section 4.2, I do not understand why max vobjective is set equal to vobjective,lb (in this way it appears as a constraint, rather than a true maximization). What is vobjective,lb?

**Response 22:** Part of the mathematical definition was missing in section 4.2 and has now been added. vobjective,lb is the lower bound of the objective rate. We have elaborated on the descriptions and refer the reader to the original publication for any further details.

2) Still in section 4.2, the models are unconstrained (except for the stoichiometry and irreversibility of course): what is the number of degrees of freedom of the different models?

**Response 23:** The degrees of freedom of each unconstrained model are now included in Table 1. They range between 14-15, not accounting for glucose and oxygen uptake rates being fixed prior to optimization.

3) the text often mentions the "excessive flexibility of FBA". I think that this statement sounds a bit too strong, and vague at the same time. Per se, FBA is an excellent tool, all depends on the degrees of freedom of the problems under considerations.
Additional information or measurements will help reduce the underdeterminacy of the problem (which is a better concept than the flexibility of a method).

**Response 24:** We agree, thank you for the feedback. In response, we have revised the manuscript to tone down the wording. E.g. the abstract and title for section 2.4 were revised and we have used data to illustrate the flexibility rather than to just state so, e.g. in the abstract: *" ..displaying up to 11-fold greater flexibility in the range of reaction fluxes than experimentally measured.."*

4) Is the CBA protocol available to the community as a Python function?

**Response 25:** yes, all python scripts has been provided in our resubmission

5) In FVA, why 99% of the maximal value of the objective function, and not 100%?

**Response 26:** The percentage was fixed at 96.77 (rounded up to 97%) as this represents the deviation in growth rates measured by Haverkorn et al. This information has been duly amended and included in the revised manuscript. For reasons we explain in **Response 16**, we switched MFA datasets and the fraction of the optimum now used is 97.2% based on the new dataset from Long et al.

6) In section 4.4, C-13 labelling experiments by Haverkorn et al. (which is reference 44 and not 46) are used to constrain the models. Afterwards, it is referred as the "Rijsewijk dataset" which is unclear. Could the authors elaborate on the fact that this dataset is appropriate to create constraints in their study?

**Response 27:** Thanks for noticing the typo which has now been corrected. When it came to picking an appropriate dataset, initially there were two landmark *E.coli* omics datasets which we could choose between. Firstly, the omics data provided by Ishii et al. (<https://pubmed.ncbi.nlm.nih.gov/17379776/>) which involved the implementation of gene knockouts directly truncating reactions within central carbon metabolism, and secondly the dataset from Haverkorn et al, a study which focused on knocking out the expression of transcriptional factors. Both of these datasets are specific to E.coli MG1655, evaluated a large number of targets and implemented a holistic omics approach to the generation, curation and analysis of the data. We initially picked the Haverkorn dataset because the knockouts were not directly influencing CCM reactions. However, this also meant that it was not possible to model the same data set with MOMA as requested by other reviewers. Hence, finally, we settled on the recent data set provided by Long et al. (2019). As far as we can tell, this is the highest quality data set available that also can be modelled.

The use of the flux values to create min-max bounds seems to be a big assumption.
What are the remaining degrees of freedom after the implementation of these constraints?

**Response 28:** Yes, the use of MFA data to constrain the model requires assumptions. Here, we approached this opportunity by empirically evaluating such an approach. As it turned out, MFA-based constraints were not able to eliminate futile co-factor cycles, nor enforce biomass as a sink, hence, we concluded that for now this is not a suitable alternative to manual elimination of futile cycles, unless any remaining cycles are manually capped. In any event, this analysis is combined also with MOMA analysis and compared against the Dugar method, and we feel that the combined analyses is worthwhile presenting to the reader. We end section 2.5 by *" We concluded that MFA-based constraints is not an ideal replacement for manual whack-a-mole futile co-factor cycle capping."*

7) Figures 3 and 5 would be easier to interpret if the same scales were used in the graphs.

Response 29: Thanks for pointing this out. We have adjusted the scales accordingly.

**------------------------------------------------------**

**Reviewer #4**: General comments:
The authors presented analyses on the cofactor balance of different production pathways for butanol and its precursors. The authors also organized the analysis steps into a workflow. By examining the energy or redox wastage the authors claimed that those flux distributions are not biologically realistic and made efforts to block such wastage like ATP hydrolysis and redox sinks and futile cycles in particular ways to find solutions in which energy and redox either go into products or growth, to predict more realistic yields. The assumption is based on previous studies that have suggested that when growth is the only major sink of energy and redox surplus, more materials (carbon, nitrogen, etc.) will be consumed by growth too and result in an overall lower yield.

Specific comments:

Though the analysis does generate some useful insight and results, and based on an assumption that is valid to a certain extent. There are major concern from the reviewer’s point of view.

Major comments

Although many observations and computations are solid, how the authors present their arguments or assumptions/hypotheses seem biased and not objectively based. And it does not represent a proper way of interpreting computational results.

1.1. One example is to directly take a certain type of FBA solutions (pFBA here) and use the flux values for intracellular reactions as the predictor and then draw conclusions from that. For example in section 2.4, the authors just take the values predicted for ATPM returned by the particular pFBA solution and claimed FBA predicted ATP wastage. The underlying philosophy of constraint-based modeling is that when we have more constraints from biochemical principles, like mass balance and reaction directionality, we can narrow down the range of phenotypes. But we should be always aware that since the system is mostly way underdetermined, we should never simply take a certain flux value and draw conclusions from that. A better way is to look at the FVA range, which the authors actually did. And from Table S1, FBA actually did not preclude solutions with minimum ATPM only (min values in all cases are 7.6). This is actually consistent with what the authors found. They were able to fix ATPM value and still get max. yields the same as before. **The authors can present the FVA ranges and then say that they include metabolic flux distributions that represent energy and redox wastage** but should not simply take a particular solution from FBA and say it predicted wastage since no wastage does lie within the prediction of FBA. It just did not exclude wastage prediction.

**Response 30:** Thank you very much for the feedback. This comment raises a highly valid and important point, to which we have made necessary amendments.

Firstly, we extended the FVA analyses, as also requested by other reviewers, and in most cases (reactions, models) there was no difference between the FVA minima/maxima and the values generated by FBA. Hence, most solutions are unique. This is described in detail above in response 1.

Secondly, we agree that it makes no sense to discuss any implications from any particular reaction flux predicted by FBA, unless there are only unique solutions. This, however, is where the strength of the CBA analysis comes through, since it considers sums of fluxes in particular categories. For example (in the case where the solutions are not unique), even though ATPM may always have an FVA minimum of 7.6 (Table S1), this minima can only be achieved (without sacrificing the optimum objective function) if another futile co-factor cycle is not active. This is illustrated by the whack-a-mole appearance of futile co-factor fluxes discussed in section 2.4. We agree fully though that we can't predict which such cycle is active and which is not, i.e. there is space of possible solutions. Since CBA only considers the sum of particular categories, such as 'ATP waste', we can address this point by making sure the discussion is mainly focused on CBA category sums rather than specific reactions, except where the flux distributions are unique, as they happened to be in many cases (as enumerated in response 1).

To address the above, we have modified the manuscript throughout, removing or replacing any comments relating to specific predicted reaction fluxes, in all cases where there are no unique solutions. Three examples here in the rebuttal illustrate the response:

(1) G6PDH2r (entry-point to PPP) and PDH only have unique solutions, whilst ATPM (as reviewer 4 noticed) does not, at least not for BuOH-0 and BuOH-1. In section 2.2 there is the following sentence "*All butanol models relied on the glyceraldehyde-3-phosphate dehydrogenase (GAPDH and pyruvate dehydrogenase reaction (PDH) for the supply of redox."* As all reaction flux predictions for both these enzymes are unique, this is a fair conclusion to make and no alterations are needed.

(2) ADK1 is variable in BuOH-0 and BuOH-1 but not in the other models, under aerobic conditions (Table S1). In section 2.2. we wrote "*As a result, we observed (1) 19.3%, 37% and 42.3% of total NAD(P)H was produced by the PPP resulting in a higher yield of NADPH per glucose and (2) activation of the ADK1 reaction to recycle AMP*". As explained above, the comment regarding PPP is fair given that G6PDH2r does not show any variation in the FVA, but, the statement regarding ADK1 is not valid under some conditions. Hence, we would adjust this sentence as follows: "*As a result, we observed 19.3%, 37% and 42.3% of total NAD(P)H was produced by the PPP resulting in a higher yield of NADPH per glucose."*

(3) In the important case of ATPM, which is variable in all the models as noted by the reviewer, we wrote "*In contrast to wild type (measured at 7.6 mmol gDW-1hr-1 as per [22]), all models displayed a flux increase through either the ATPM or ADK1 reaction of up to 3-fold,* ***ranging*** *between 9-25 mmol gDW-1 hr-1 under aerobic conditions (Table S1).* ***Up to*** *71.4% of the total ATP produced was dissipated through this reaction alone, suggesting surplus energy in many of the models.".* Here, we refer to the possible range shown in the FVA data (the reference to Table S1 has been added), and emphasise that it is a range by adding "Up to..". What we should have also done, however, is to point out that also other ATP-burning reactions could substitute for the ATPM reaction, as summed up by the ATP "waste" and "maintenance" CBA categories shown in (now) Figure 4A. In part, this is due to the fact that the other reactions are the main focus of the next section. To clarify this, we added references to other reactions and section 2.4 (in section 2.3), as follows. From "*As the ATP demand of the synthetic pathways increased, the fraction of ATP wasted by ATPM also gradually dropped, Figure 4A (blue)."* was revised to "*As the ATP demand of the synthetic pathways increased, the fraction of ATP wasted by ATPM****, or other ATP-burning futile cycles (Fig. 5A),*** *also gradually dropped (Figure 4A).*"

In any event, it is also worth pointing out that manual curation tries to address the presence of futile cycles, which in fact happen as a result of the flux variability through co-factor related reactions (Table S1). After manual curation, the models led to unique solutions in all cases (Tables S9 and S10), in which case this would no longer be an issue.

1.2. Following the last comment, lines 192 – 194 claims ‘Five out of eight models surprisingly had the same theoretical maxima under aerobic conditions achieved through considerable adjustments to the network flux distributions’. But the authors looked at only one solution using pFBA for each model. The possibilities that there are other solutions that are more similar to each other given the same yields cannot be ruled out. The authors should claim that only if they have tried to, for example, add the maximum yields as constraints and minimize the difference (in whether a reaction is used, or just sum of squared difference) between all models and still see that they are significantly different.

**Response 31:** We interpret this statement referred to the **maximum** possible yield reached for BuOH-0, BuOH-1, CROT, BUTYR and BUTAL., as shown in the first version of Figure 1, which has now been updated to show carbon yields. The statement was not an important observation and the corresponding sentence has now been removed after we revised the document to make it more concise.

Also, from Table S2, only BuOH-0, BuOH-1 have the same max. of 10 mol/10 mol glucose, while tpcBuOH, BuOH-2 and fasBuOH have lower yield. Did the author include also CROT, BUTYR and BUTAL which produce different end products (though they are precursors of butanol)? But still they are not quite comparable especially they involve cofactors that directly impact the final yield.

**Response 32:** We assume this comment is related to that above. The "case study" models employed were 5 pathways leading to n-butanol and three pathways leading to other end-products in the same pathway, in order to create variation in both electron and ATP demand. Table S2 does include also the CROT, BUTYR and BUTAL models, but the reviewer is correct that the sentence could have been reworded to say that 2 out of 5 butanol models had the same maxima, and group the butanol precursor models separately.

2. Line 303 asks “Is bacterial metabolism that flexible”? The authors did not directly answer this but from the analyses afterward it is pretty apparent that their answer was ‘NO’. But the reviewer does not see very strong evidence about that. The reviewer hopes that this is a misunderstanding originating from how the authors interpret the flux distributions from FBA. From the text later, the reviewer could see that the authors argued that it is not possibly that flexible because there is energy and redox surplus that go into wastage sink or futile cycles which are not biologically realistic. The authors argue that they should go into growth. So growth must accompany the product formation. Well, that should be obvious enough for most metabolic engineers. No growth no nothing no matter the product generates energy or redox surplus. But mostly metabolic engineers look at theoretical yield because we hope that the pathway can be active under the condition of growth or after growth. So under growing conditions, is that possible for the bacteria to include very different pathways with different cofactor balance profiles to the product formation? The reviewer tends to say yes. Just that the absolute fluxes through the pathways could be different depending on how well it is coupled to growth. Even not under growth conditions, the reviewer does not see the reason why those pathways are necessarily not feasible. Since they are engineering pathways anyway, there could be engineering efforts to further improve that. For example there have been work to introduce ATPase activity to consume ATP in resting cells and observed enhanced fluxes.

**Response 33:** This comment relates to the question of flexibility. We agree it is difficult to completely answer the question of whether bacterial metabolism is as flexible as the analyses (mainly highlighted in section 2.4) suggest. But we feel it is an important topic to discuss in relation to computational modelling. We include several examples from the literature (final paragraph 2.3; 2nd paragraph, 2.4) that in many cases are based on experimental laboratory measurements, including metabolic engineering (e.g. PYK, PPC). Although difficult to measure, most such references cited reach similar conclusions, bacterial metabolism has evolved to be tightly regulated and since stoichiometric models don't account for regulation, it makes sense that the answer to the question is 'no'. As pointed out by the reviewer, numerous studies on the effect of artificial ATP-metabolism on product yield have been carried out and should have been included also in this article. We now added one such recent study (Boecker et al, 2019; <https://doi.org/10.1002/biot.201800438>) as an example in section 2.3.

" *The observation that artificially enforced ATP-hydrolysis can enhance product yield with engineered E. coli (Boecker et al, 2019) supports the idea that ATP availability influences the allocation between biomass and other carbon-products."*

3. The argument in Lines 336 – 342 for “FBA having more flexibility than what would be expected in reality” seems highly speculative. Is the ‘flexibility’ that the authors were referring to the same as the ‘flexibility’ that ref. 27 (Ghosh, PLOS ONE, 2011)? It seems that in that article restoring the ‘flexibility’ is something desirable for the cells while here the authors were saying such flexibility was not realistic. There seemed to be some misinterpretation without proper definition. By the way the reference for ref. 27 needs fixing.

**Response 34:** As reviewer #3 pointed out in response 3, as well as reviewer #4 in response 30 this excess “flexibility” observed in FBA occurs as a result of the underdeterminacy of the system, which enables FBA to readjust the fluxes. The effect of constraints is further experimentally evaluated in section 2.4, and the relationship between measured and predicted fluxes is discussed in section 2.6. A definition to the first use of "flexibility" in this context was added to the end of section 2.2, and we hope it should now be clear what we are referring to.

"*We asked ourselves, is bacterial metabolism really this flexible? I.e. is the range of flux solutions predicted by stoichiometry-based modelling greater than what is possible in reality?"*

4. Lines 345-346 are misleading. Since the authors only used the E. coli core model, which does not have glycerol in the model, of course the solutions cannot resort to glycerol production. And it is just very intuitive that FBA will simply not produce biomass to consume the energy or redox in excess. Biomass production competes cellular resource with the pathways for producing many compounds because in addition to energy or redox, biomass production involves the synthesis of many metabolites and producing any biomass will very probably reduce the maximum yield of butanol, contradicting to the assumption of optimal yield when the authors ran pFBA.

**Response 35:** The comment regarding Dugar's results were phrased as "..such solutions.." and we are confident that readers will understand that we don't specifically refer to glycerol only. Although the biosynthesis of biomass is wasteful from a computational perspective, it competes for resources (from a biotechnological perspective) in reality. That is why Dugar et al. made the assumption that excess ATP in a pathway will result in biomass formation. As we demonstrate in section 2.4, once the artificial unrealistic co-factor cycles have been capped, biomass synthesis is predicted to deal with excess ATP availability.

The comment *"Left to its own devices, FBA did not resort to such solutions with the core wild-type model*" is referring to a comparison between FBA and the Dugar model in the sentence above, with the aim to lead in to the next section 2.4, where manual constraints are used to minimise futile cycles resulting in biomass accumulation.

5. Section 2.7 comparing the ranges from FBA and MFA could be over-/mis-interpretation of data. Metabolic fluxes are almost never directly measured (not that the reviewer knows). Flux ratios between pathways are usually first measured (this point included the assumption of which pathways to include) and then fitted into a stoichiometric model (usually constructed manually to have a degree of freedom <= 0 after constrained by the measured flux ratios). Therefore the observation that MFA flux ranges are narrower than FBA ranges by direct comparison are not necessarily valid because the models used to interpret the data or do predictions are different, which impact the results a lot (or at least to an unknown extent). Though I think the authors’ conclusion of MFA narrowing down FBA ranges must be correct, the reason is simply because MFA flux ratios give additional information that an FBA model does not have by default. But comparing directly this way without any elaboration gives a biased understanding. Did the authors examine carefully the original stoichiometric model used for fitting the flux data and check that it still largely applies to the E. coli core model? Or just fit the data into the current model to check potential range?

**Response 36:** Thank you for the comment - this is now section 2.6 in the revised manuscript. We have considerably amended this section, following suggestions by reviewer #1 (response 16) and we have now also added MOMA-based analysis. Arguably, the MFA-part of this section is based on a number of assumptions regarding 13C-MFA. In the absence of further information, and given the large body of literature on the topic of 13C-based metabolic flux analysis, we believe it is fair to assume that the 13C-MFA data is at least representative of intracellular fluxes in central carbon metabolism and therefore can be used for the purpose of this study. The aim is to understand whether there is a difference in the predicted, measured and fitted flux variability of bacteria, and to address the question of whether additional constraints may eliminate the need for manual capping of artificial futile cycles, or not. Unfortunately, we conclude that the use of 13C-MFA data as constraints does not resolve this issue, but we feel it was valuable to try.

Besides the interpretation of computation results, there are some major concerns in the computation methods used.

6.1. Related to the first point, the CBA algorithm only analyzed a particular solution from FBA. It is not a systematic analysis. There was a previous study introducing a technique called flux-sum analysis (Chung and Lee, BMC Syst. Biol., 2009, <https://bmcsystbiol.biomedcentral.com/articles/10.1186/1752-0509-3-117>) to address this issue. Basically one should find the ‘cofactor turnover’ range by minimizing and maximizing the sum of all cofactor score (as defined by the authors) to fully describe the flux through a cofactor. Similarly, for the energy and redox balance profile breakdown, one should minimize and maximize the sum of all cofactor score across reactions belong to a particular category and present a range. Simply comparing one particular flux distribution from each model is not representative.

**Response 37:** This comment is not entirely clear. As explained in responses 1 and 30, the majority of the solutions studied in this article are unique. We appreciate that better methods may exist, but the CBA method used in this article is based on summing reactions belonging to key categories. Even though more than one solution may exist for certain reactions, at least in the crucial category of ATP "waste", it is the sum given by CBA that is more important than any one individual reaction as it provides the key information useful for a metabolic engineer, i.e. most pathways studied in this article have an excess of ATP.

6.2. Why did the authors define CO2-releasing NADH-producing reactions such as PHDc as waste? It is hard to understand. Looking at the right plot in Figure 3B, a large portion of the ‘NADH waste’ (+ve) does go into ‘NADH target’ (-ve). Is that a proper definition of waste?

**Response 38:** The desired commercial products are carbon-based, hence we define all reactions that result in a loss of carbon as "waste", incl. CO_2_, namely due to the fact that it is not just the reaction itself we evaluate, we evaluate the flow of co-factors and what these are doing, e.g. it NAD gets reduced to NADH at the expense of CO2 release, that will be considered waste for the purposes of producing such NADH. Alternative categorization and labelling is also possible but we settled on the current method illustrated in Figure 2 as we feel it is useful for metabolic engineers.

7.1. The procedure to eliminate futile cycles described in lines 372 to 384 is problematic. Bounding the fluxes with the values from the wild-type flux distribution, which was obtained by maximizing biomass production, will probably make the predicted fluxes look like the growth metabolism. This also potentially excludes other reactions that can be used but not active under the condition of maximum biomass production.

**Response 39:** We agree that this choice is not perfect, but it represents a reasonable compromise for a first proof-of-concept as it still allows for biomass synthesis (essential to synthesise the biocatalyst and why a non-zero ATPM is maintained), and indeed when the artificial co-factor cycles were minimised, new cycles appeared, and eventually biomass was the only final sink for excess ATP, thereby connecting the present study with the original work (Dugar et al) where biomass was assumed to act as a sink for excess ATP. As we mentioned previously, in reality, biomass and bioproduction compete for resources, so there will always be a trade-off between growth and bioproduction metabolism, so we believe that the approach and outcome are a fair compromise of what we are to expect also in experimental conditions.

7.2. There are well-established techniques to add constraints to exclude all futile cycles while not excluding any feasible flux distributions without futile cycles. For example, adding integer cuts iteratively instead of adding bounds on specific reactions. More systematically, one can use the loopless FBA formulation (first published in Schellenberger et al., Biophysics Journal, 2011 and a number of performance improved formulation later). The formulation usually blocks completely balanced cycles which has delta G = 0. For the purpose here, one can simply calculate the null space which excludes the cofactors of interest (ATP, ADP, Pi, H2O, H+, NADH and NAD+) and use the same formulation accordingly. Then all cycles whose net effect are consumption or production of the cofactors will be prohibited in the solution. See the Methods section in Ng et al., Scientific Report, 2019 (<https://www.nature.com/articles/s41598-019-38836-9>) for an example. Basically you just equations (6) - (10) in the method section

**Response 40:** Thanks for the suggestion. We appreciate that many other methods could be applied to the broader subject of investigation in this manuscript, however, the more the storyline diverges the more it turns into a new paper. Hence, we would prefer to constrain the scope of the present article, and this would also avoid wasting the effort of the three other reviewers.

Nevertheless, as a test we implemented loopless FBA with the butanol producers, using the COBRApy "loopless_solution" function, which the COBRApy documentation specifically recommends to use instead of the COBRApy algorithm adapted from Shellenberger et al. The results have been submitted in an .xlsx file as part of the Supplement. Unfortunately, the loopless FBA function did not prevent the whack-a-mole appearance of futile co-factor cycles. We added a description of this to the abstract and first paragraph of section 2.6.

8. Why did the authors only look at the E. coli core model? As stated in comment 4, that potentially causes some systematic errors because a genome-scale E. coli model could probably predict energy or redox consumption through glycerol production but the current model just has no such reaction. Does the argument still hold in general?

**Response 41:** The curated *E. coli* core model is a well-established simple model that has been used by many others and it could be argued that is the most accurate model available given that increasingly complex models shift the focus away from central metabolism and are more likely to also incorporate reactions that do not reflect reality. Indeed, the *E. coli* core model lacks some of the metabolic features in the yeast system evaluated by Dugar et al., but we don't agree that this would cause systematic errors that reduce the value of the study in *E.coli* and the principles evaluated here should be applicable also to other larger more complex models. Whenever a new concept is evaluated we would argue that it makes most sense to commence with the simplest most well-understood system available.

9. Overall, the reviewer does think the authors had some useful ideas presented but the way of presentation and interpretation is somehow biased and the computational method is not systematic enough to draw proper conclusions. The reviewer suggests that the authors state clearly their assumptions in exact terms. The wording in the abstract and introduction is not clear. Not until in the middle of the results the reviewer could understand what the issues they were trying to address. Simply state that energy and redox surplus associated to a certain engineering pathway should be consumed only by growth but not artificial sinks in the model (ATPM, etc.) and futile cycles which are tightly regulated. So the authors developed a method to first check the RANGE of energy and redox balance profile (by doing the flux-sum analysis with reactions categorized into sets for growth, product, waste, etc.) to see if a pathway has energy or redox surplus. Then formulate an optimization method based on loopless FBA for predicting pathways and theoretical yields in the absence of unconstrained high wastage flux and futile cycles. For the MFA constraint added, the best would be to add the originally derived flux ratios as constraints. If not possible at least the assumptions or applicability should be carefully studied and discussed. The reviewer would appreciate in the more general and systematic effort to find out pathways that couple growth to energy and redox surplus to have a more realistic yield and pathway prediction.

**Response 42**: The article has now been comprehensively revised, including revision of both the abstract and introduction. Key assumptions are now mentioned in both the abstract and introduction. The focus of the work still remains the same, i.e. to develop an FBA-based approach to co-factor analysis of metabolic engineering designs, thereby extending the previous work of Dugar et al. The changes recommended by the reviewer are of interest but we would suggest that they form the basis of new studies in the future that build upon this, previous work by Dugar et al., and the other papers mentioned by the reviewer.

Minor comments:

- Please include the gene/reaction names in Figure 1. It should not difficult to explicitly show and name all eight different routes with good use of colors. Or beside the main figure, add thumbnail versions of all different pathways without the detailed names but simply arrows for the particular routes

**Response 43:** Thanks for the suggestion. Figure 1 has been revised accordingly.

- Refer to Tables S2 and S3 for the FBA-calculated yields between line 185 and line 188.

**Response 44:** Agreed and implemented.

- What does the % yields between lines 185 and 188 mean? They are neither weight nor carbon yield. If it is simply molar ratio of butanol to glucose, that should not be reported as a percentage. Like probably no one will say the theoretical yield of glycolysis from glucose to pyruvate is 200% just because 1 mol of glucose yields 2 mol of pyruvate. The reviewer suggests using carbon yield instead, i.e., 1 mol glucose to 1 mol butanol means 66.67% carbon/carbon yield. The same goes for Figure 3A.

**Response 45:** Thank you, this is a good idea. The reported values in l.193 and l.196, and throughout the whole manuscript, have been amended to now be given as carbon yield.

- Line 279: please state the reaction name and ID corresponding to Tables S4 or S5.

**Response 46:** Thank you, amended.

- Line 291: which figure the authors were referring to? Figure 3B or 3C, ATP or NADH?

**Response 47**: This statement was removed in the revision of the manuscript in response to other reviewer comments and a general clean-up. It referred to Fig. 3A and B.

- Lines 293 and 295: it reads incorrect to the reviewer to call PFL and PDH the same/identical reaction. In particular for these two reactions. The analysis can be made more relevant by turning on PFL only in anaerobic conditions, which is a consensus on PFL activity.

**Response 48:** Agreed, thanks for noticing this mistake and the lack of clarity. The statement has been amended as follows: *“Further differences in the CBA redox profiles may arise from the fact that flux may or may not be directed via co-factor-dependent routes (e.g. PFL (electrons channeled into H_2_ or excreted formate under anaerobic conditions) vs. PDH (electrons channelled back into NAD^+^ as per Tables S2 and S3, Supplement)..”*

- Section 2.3 is not well-oriented. Not until the very last sentence only the reviewer can get the message that the authors were trying to assess how practical/biologically realistic the solutions from pFBA are. Please include a topic sentence telling what the authors want to examine and what they did before going deep into the details. For example, saying the following in the beginning of that paragraph will orient the readers much better (a pretty arbitrary construction that does not seems right): ‘Since realistic bacterial metabolism usually exhibits only a limited flexibility [if that is the assumption the authors have], we examined the plasticity in the FBA solutions for different pathways to determine if they together fit into a realistic description of bacterial metabolism.’

**Response 49:** There may be some misunderstanding here, or is it possible that the comment refers to another section? The cited statement could not be found either.

The aim of section 2.3 was not to evaluate how realistic pFBA solutions are, rather this is the question we ask following the analysis presented in this section, and to link section 2.3 to 2.4. The aim of section 2.3 is to present the CBA tool as we built it: how it works, the kind of results that it produces, and a deeper analysis of these results. It is as a result of this analysis that we notice the issues with underdeterminacy, and the extent to which co-factors may be wasted to set the solution into balance, hence why we move on to evaluate this question of how realistic the estimates are in section 2.4. Upon revising the manuscript, we have tried to make sure this flow is more explicit to the reader.

- The text in lines 312 (“all models displayed a flux increase through the ATPM reaction …”) and 324 (“models tpcBuOH, BuOH-2 and fasBuOH did not show any energy surplus”) and the data in Table S2 (ATPM = 7.6 for WT, tpcBuOH, BuOH-2 and fasBuOH) seem to be contradictory.

**Response 50:** We agree, thanks for noticing this contradiction. Models tpcBuOH, BuOH-2 and fasBuOH do not consume any surplus ATP through the ATPM reaction (as the reviewer points out, ATPM’s flux is 7.6 in these models). However, the surplus is instead consumed by the ADK1 reaction, thereby recycling the excess AMP produced via the CAR-dependent butanol-pathways. We have clarified this and removed the contradiction in the manuscript.

- The math and the algorithm are not clearly presented in the Methods section (4.2) and not in Figure 8. For example, CFS = co-factor stoich. x flux should be written using the standard symbols which have already been introduced in the FBA section, for example:
o CFS_i,j = S_{i,j}v_j for cofactor i in reaction j.
o CFS_i = \sum_{reactin j, if CFS_i,j > 0} CFS_i,j, or simply \sum_{j} abs(CFS_i,j)/2 as in the flux-sum analysis paper defined (Chung and Lee, BMC Syst. Biol., 2009, <https://bmcsystbiol.biomedcentral.com/articles/10.1186/1752-0509-3-117>

**Response 51:** Thank you. Amended.
